# On the Origin and Evolution of the Material in 67P/Churyumov-Gerasimenko

**DOI:** 10.1007/s11214-020-00718-2

**Published:** 2020-07-30

**Authors:** Martin Rubin, Cécile Engrand, Colin Snodgrass, Paul Weissman, Kathrin Altwegg, Henner Busemann, Alessandro Morbidelli, Michael Mumma

**Affiliations:** 1 Physikalisches Institut, University of Bern, Sidlerstrasse 5, 3012 Bern, Switzerland; 2 CNRS/IN2P3, IJCLab, Université Paris-Saclay, 91405 Orsay Cedex, France; 3 Institute for Astronomy, University of Edinburgh, Royal Observatory, Edinburgh, EH9 3HJ UK; 4 Planetary Science Institute, Tucson, AZ 85719 USA; 5 Institute of Geochemistry and Petrology, Department of Earth Sciences, ETH Zurich, Zurich, Switzerland; 6 Observatoire de la Cote d’Azur, Nice, France; 7 NASA Goddard Space Flight Center, 8800 Greenbelt Rd., Greenbelt, 20771 MD USA

**Keywords:** Comets, Origin of solar system material, Rosetta mission, 67P/Churyumov-Gerasimenko, Icy bodies, Isotopic and molecular composition

## Abstract

Primitive objects like comets hold important information on the material that formed our solar system. Several comets have been visited by spacecraft and many more have been observed through Earth- and space-based telescopes. Still our understanding remains limited. Molecular abundances in comets have been shown to be similar to interstellar ices and thus indicate that common processes and conditions were involved in their formation. The samples returned by the Stardust mission to comet Wild 2 showed that the bulk refractory material was processed by high temperatures in the vicinity of the early sun. The recent Rosetta mission acquired a wealth of new data on the composition of comet 67P/Churyumov-Gerasimenko (hereafter 67P/C-G) and complemented earlier observations of other comets. The isotopic, elemental, and molecular abundances of the volatile, semi-volatile, and refractory phases brought many new insights into the origin and processing of the incorporated material. The emerging picture after Rosetta is that at least part of the volatile material was formed before the solar system and that cometary nuclei agglomerated over a wide range of heliocentric distances, different from where they are found today. Deviations from bulk solar system abundances indicate that the material was not fully homogenized at the location of comet formation, despite the radial mixing implied by the Stardust results. Post-formation evolution of the material might play an important role, which further complicates the picture. This paper discusses these major findings of the Rosetta mission with respect to the origin of the material and puts them in the context of what we know from other comets and solar system objects.

## Introduction

Comets are the least processed objects in the solar system. Studying them allows us to gather precious information about the early days of our solar system and possibly even beyond. The origin and processing of the material also holds important clues to our understanding of the formation of our solar system some 4.6 Ga ago. Early remote sensing observations of the anomalous acceleration of comets Encke, D’Arrest, and Wolf 1 led Whipple (1950, 1951) to the conclusion that comets are “dirty snowballs” emitting gas and dust which can either decelerate or accelerate the comet along its orbit around the sun. However, only in 1986 did the European Space Agency’s (ESA) Giotto mission and the Soviet Vega 2 mission flying by comet 1P/Halley (Reinhard 1986) confirm that comets possess a solid nucleus composed of volatile and refractory materials. From these investigations Geiss (1987) established that comets have preserved the accreted and condensed materials better than other objects in the solar system.

One of the main goals of space missions to comets, and dedicated remote sensing campaigns, is to understand their environment and to link their composition to the early days of the solar system. Several spacecraft have since visited comets *in situ* or even brought back a sample: Giotto also flew-by comet 26P/Grigg-Skjellerup (Coates et al. 1997), Deep Space One passed by 19P/Borrelly (Soderblom et al. 2002), and 81P/Wild 2 was visited by the Stardust spacecraft before its extended mission, NExT, flew by 9P/Tempel 1 (Brownlee et al. 2006). Cometary dust from Wild 2 was brought back to Earth for in-depth analysis. Additional comets visited were 9P/Tempel 1 by Deep Impact (A’Hearn et al. 2005) and 103P/Hartley 2 by the (renamed) EPOXI spacecraft (A’Hearn et al. 2011), before ESA’s Rosetta mission encountered comet 67P/C-G (Glassmeier et al. 2007). Furthermore numerous spectroscopic and photometric observation campaigns of cometary comae have been performed from Earth- and space-based telescopes (see e.g. Biver et al. 2002; Bockelée-Morvan et al. 2004; Mumma et al. 1996, 2003, 2005; Dello Russo et al. 2007, and Lis et al. 2019). These observations have greatly increased the sample of investigated comets and led to many discoveries and allowed for a detailed comparison between comets, other solar system objects, and all the way to the interstellar medium.

Lately the Rosetta mission to comet 67P/C-G came to its conclusion. One of the main tasks of the mission was to obtain relevant measurements in both the refractories and volatiles to address the origin of the material in 67P/C-G’s nucleus. There are two main scenarios for the origin of the material incorporated into comets that are widely discussed in the community. First the inheritance from the interstellar medium (Greenberg 1982) where the chemistry occurs at very low temperatures (<20 K) driven by cosmic rays and UV radiation and/or on the surface of icy grains. The alternative scenario is the formation together with the solar system but in its cold, outer regions (Lunine and Stevenson 1985; Mousis et al. 2016a). The presence of crystalline silicates was first determined in comet 1P/Halley (Bregman et al. 1988; Campins and Ryan 1989). Analysis of IRTF spectra (Hanner et al. 1994) and later observations with the Infrared Space Observatory (ISO) (Wooden et al. 1999; Crovisier et al. 2000, and Wooden et al. 2004) suggested that a large fraction of the cometary minerals formed at high temperature in the inner solar system. The formation and incorporation of these minerals in comets thus required models for large scale mixing in the protoplanetary disk, as proposed by several authors (e.g. Bockelée–Morvan et al. 2002; Ciesla 2007). The refractory samples returned from comet 81P/Wild 2 by the Stardust mission confirmed that the comet contains at least 10% by mass of material that formed in the hot inner regions of the protosolar disk (Brownlee et al. 2006). However, the cometary volatile abundances observed in comet C/1995 O1 (Hale-Bopp) showed striking similarities to the volatile molecules from the cold interstellar medium (Bockelée-Morvan et al. 2000). Moreover, comparisons of HCN and NH_3_ with C_2_H_6_ and H_2_O amongst 30 comets suggested that both HCN and NH_3_ were better associated with C_2_H_6_ production rather than H_2_O, and both showed enrichments in comets within 1 au of the Sun consistent with dissociation of the semi-volatile salt, ammonium cyanide (Altwegg et al. 2020; Hänni et al. 2019; Mumma et al. 2017, 2018, 2019). Evidently, multiple processes are at work, although their relative importance may differ from not only one comet to another but also among the various molecules. Furthermore, post-formation alteration of the ices in comets has to be taken into account, in particular the various heating processes and associated outgassing of species of high volatility. Critical information is thus found in a combination of the elemental, molecular, and isotopic abundances of volatile and refractory material in comets by comparison to the solar system bulk composition, other comets, and interstellar medium abundances.

In this paper we will discuss how the material found in comets and in particular 67P/ C-G can be traced back to its origin (see also Levasseur-Regourd et al. 2018). Furthermore we refer to the paper by Weissman et al. (2020) on the formation and dynamical history of cometary nuclei.

## Observation of Comets

The low level of evolution of comets makes them ideal targets to study the history of the material in our solar system. The details of the ices in a comet’s nucleus are imprinted in the gas coma surrounding it and are thus accessible not only to *in situ* but also to remote sensing observations.

### Abundances of Volatile Species in Comets and Classification

The composition of comets, and its variability among the different dynamical groups, is a crucial link to understanding the formation of our solar system. Remote observations measure composition via spectroscopy or narrowband photometry, with a long history in the ultraviolet and blue visible wavelength range, where emission bands from ‘daughter’ species such as OH, CN, and C_2_ are seen. More recently, there are increasingly observations in the infrared and at sub-mm wavelengths, which are sensitive to rotational and vibrational excited modes in ‘parent’ molecules (Bockelée-Morvan and Biver 2017), and with space telescopes that can directly measure the most abundant trace species (CO_2_), which cannot be observed through Earth’s atmosphere, and other gases that require a significant Doppler shift for ground-based detections (e.g., CH_4_, CO).

**Fig. 1:**
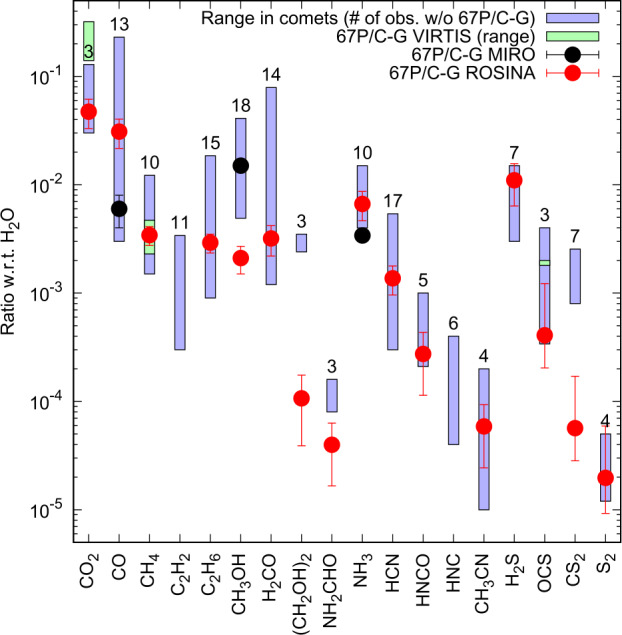
The blue boxes represent the observed range of volatile species w.r.t. H_2_O with at least the indicated number of detections in comets (aside 67P/C-G) within 2 au from the Sun (Biver et al. 2015; Bockelée-Morvan et al. 2004 and references therein). Separately listed are relative abundances of 67P/C-G from Table 2 measured by VIRTIS (range given by green boxes; Bockelée-Morvan et al. 2016), MIRO (black; Biver et al. 2019), and ROSINA (red, different isomers may be present, e.g. HCN cannot be distinguished from HNC with ROSINA; Rubin et al. 2019a)

Figure 1 shows in blue boxes the abundance range of commonly observed volatile species with respect to water in comets. Shown is a selected set of species detected in at least 3 comets aside from 67P/C-G. The most abundant species are H_2_O, CO_2_, and CO. Lower abundances are found for other CH- and CHO-bearing molecules and even lesser amounts for the CHS- and CHN-families of volatile species (Bockelée-Morvan et al. 2004; Dello Russo et al. 2016). These volatile species cover a wide range of sublimation temperatures and therefore relative abundances in the coma depend on the heliocentric distance. For comparability the observations in Fig. 1 are therefore limited to <2 au.

The first assessment of the average composition of a large sample of comets, based on narrowband photometry, was published by A’Hearn et al. (1995), who identified two broad taxonomic classes, ‘typical’ and ‘carbon-chain depleted’, the latter having lower production rates of C_2_ and C_3_ (relative to CN), and origins in the Kuiper belt. More recent work covering comets over a wider range of heliocentric distance has shown that these production rates vary significantly depending on where in its orbit the comet is observed (Opitom 2016), suggesting that this taxonomy must be used carefully. An updated review of the abundances of the major volatiles (CO, CO_2_, H_2_O) by A’Hearn et al. (2012), including the dedicated campaign for the CO_2_ abundance in 18 comets by the Akari spacecraft (Ootsubo et al. 2012), found no evidence for significant bulk compositional variation between comets from the Kuiper belt or the Oort cloud.

Our knowledge of the minor constituent ices in comets primarily comes from relatively bright comets that can be observed at longer wavelengths (infra-red to radio), with many species first identified in observations of the great comets Hyakutake and Hale-Bopp (Bockelée-Morvan et al. 2004). Telescope technology has advanced sufficiently in the decades since these comets that there are now sufficient observations to attempt to group comets based on abundances of relatively rare parent molecules (e.g. hydrocarbons, methanol, and nitrogen-bearing compounds), with a general picture of greater abundance of more volatile ices in Oort cloud comets (Dello Russo et al. 2016; Mumma and Charnley 2011), thought to be less processed, but with an uncertain influence of the different evolution of short and long period comets (SPCs and LPCs). The utility of such taxonomies is still debated however, and no clear definitions have been broadly accepted; there is a very large diversity of relative abundance of different species among comets, with variation over orders of magnitude (Fig. 1; cf. Biver and Bockelée-Morvan 2015). An emerging view has some volatiles produced at least in part from semi-volatile ammonium salts (e.g., $\text{NH}_{4}^{+}\text{CN}^{-}$) (cf. Altwegg et al. 2020; Hänni et al. 2019; Mumma et al. 2019; Poch et al. 2020; Quirico et al. 2016).

High-resolution spectroscopy of brighter comets can also reveal compositional variation at an isotopic level (see Sect. 4); very recent results suggest differences in the isotopic composition between ‘hyperactive’ comets and those with more typical water production rates (Lis et al. 2019), in this case pointing to observed differences due to the current evolutionary state of the comet rather than its origin location. Finally, ground-based observation of large numbers of comets still reveals surprising cases with very unusual composition (e.g. the N_2_-rich comet C/2016 R2; Biver et al. 2018; McKay et al. 2019; Opitom et al. 2019), or variation in relative abundance of different species with heliocentric distance (e.g., C/2009 P1; Feaga et al. 2013; Gicquel et al. 2015).

### 67P/C-G Abundances of Volatiles and Refractories

Ground-based observations indicated that 67P/C-G likely belongs to the carbon-chain depleted category of comets (Schulz et al. 2004), but *in situ* measurements were required to get a more detailed picture: despite a large campaign of observations supporting the Rosetta mission (Snodgrass et al. 2017), only the brightest emission features were detectable in remote data, and then only around the perihelion period while the Southern hemisphere was illuminated (Opitom et al. 2017; Snodgrass et al. 2016). Abundances of the major volatiles at comet 67P/C-G have been obtained by the Rosetta science instruments: ROSINA (Rosetta Orbiter Spectrometer for Ion and Neutral Analysis; Balsiger et al. 2007), VIRTIS (Visible and InfraRed Thermal Imaging Spectrometer; Coradini et al. (2007)), MIRO (Microwave Instrument for the Rosetta Orbiter; Gulkis et al. 2007), Alice (Stern et al. 2007) and the two mass spectrometers Ptolemy (Wright et al. 2007) and COSAC (Cometary Sampling and Composition experiment; Goesmann et al. 2007) on the Rosetta lander Philae. Most comet mission encounters lasted only for a short time, but ESA’s Rosetta mission accompanied comet 67P/C-G for over two years along its orbit around the sun, accessing a much more detailed picture of the composition and activity of the comet.

A collection of volatile abundances measured with ROSINA are found in Table 1 (Bieler et al. 2015a; Le Roy et al. 2015). These measurements were obtained *in situ* and inbound from 3.1 au and are separated between the northern and southern hemispheres of the comet. Due to the axial tilt of the rotation axis of 52^∘^ with respect to its orbital plane (Sierks et al. 2015) the comet is subject to strong seasonal differences in the outgassing (Hässig et al. 2015). The northern hemisphere was in summer for the period of the measurements in October 2014 and thus dominated total outgassing. These early observations are compared to the relative abundances from the COSAC mass spectrometer on the Rosetta lander Philae (Goesmann et al. 2015). The measurements were obtained on 12 November 2014 at 3.0 au after the initial touchdown and rebound on the Agilkia landing site, some 25 minutes into the flight towards the final touchdown location in Abydos. The mass spectrometer Ptolemy was in operation during the same time; Wright et al. (2015) reported −CH$_{2}{-}$ and −O− bearing compounds without providing relative abundances. The findings include the potential presence of polyoxymethylene (POM), a radiation-induced polymer, while other species, such as aromatic hydrocarbons including benzene and sulfur-bearing species, were either absent, or, in the case of nitrogen-bearing species, very low in abundance.

**Table 1:** Subset of volatile species in the coma of comet 67P/Churyumov-Gerasimenko measured by in situ mass spectrometry ≥3 au by number (in % normalized to water). ROSINA measurements were obtained above the southern and northern hemispheres on 19 October 2014 and 20 October 2014, at approximately 10 km from the nucleus, respectively. References: Le Roy et al. (2015) and Bieler et al. (2015a). COSAC measurements from 12 November 2014, some 25 minutes after the initial touchdown at Agilkia on the way to Abydos, are likely a mixture of coma volatiles and excavated comet material from the initial landing (see the corresponding references for the full list of molecules). Note that for some molecules different isomers have been reported, e.g. HNC cannot be distinguished from HCN. Ptolemy: see text

Molecule	ROSINA w.r.t. H_2_O [%]		COSAC w.r.t. H_2_O [%]
	North	South	Philae/Agilkia
H_2_O	100	100	100
CO_2_	2.5	80	
CO	2.7	20	1.2
O_2_	3.8	3.8	
CH_4_	0.13	0.56	0.5
C_2_H_2_	0.045	0.55	
C_2_H_6_	0.32	3.3	
CH_3_OH	0.31	0.55	
H_2_CO	0.33	0.53	
HCOOH	0.008	0.03	
(CH_2_OH)_2_	0.0008	0.0025	0.2
HCOOCH_3_	0.004	0.023	0.4
CH_3_CHO	0.01	0.024	0.5
NH_2_CHO	<0.0001	<0.001	1.8
NH_3_	0.06	0.15	
HCN	0.09	0.62	0.9
HNCO	0.016	0.031	0.3
CH_3_CN	0.006	0.016	0.3
HC_3_N	<0.00002	<0.0005	
H_2_S	0.67	1.75	
OCS	0.017	0.098	
SO	0.004	0.0014	
SO_2_	0.011	0.041	
CS_2_	0.003	0.024	
S_2_	0.0004	0.0013	

A direct comparison of the relative abundances is difficult as the COSAC and Ptolemy mass spectra contain a mixture of coma volatiles and excavated surface material from the initial landing. Therefore, Altwegg et al. (2017b) compared the results of the two lander mass spectrometers with a dust impact event that occurred in ROSINA DFMS on September 5, 2016. As a result, the previously reported presence of methyl isocyanate (CH_3_NCO), propanal (C_2_H_5_CHO), and glycol aldehyde (CH_2_OHCHO) from COSAC was not supported. The signal of POM in Ptolemy, on the other hand, was attributed to toluene and hence an aromatic hydrocarbon, previously thought to be absent. Nevertheless, all three instruments together revealed a chemical complexity of the organics in, on, and around 67P/C-G that is much greater than expected.

Table 2, for comparison, lists relative abundances of a similar set of species but measured closer to perihelion or, when indicated, integrated over the whole Rosetta mission and hence dominated by the peak outgassing period around perihelion. *In situ* measurements by ROSINA were obtained at the end of May 2015, before perihelion passage (13 August 2015 at 1.24 au). This period, when Rosetta was passing rather closely over the then-active southern summer hemisphere and outbursts were still limited (Vincent et al. 2016), was identified by Calmonte et al. (2016) to be suitable to estimate bulk abundances. This approach assumes that the high erosion rate of the comet leads to relative abundances of the gases in the coma that reflect the composition of ices inside the nucleus. Table 2 also lists a suite of relative abundances measured by the Rosetta remote sensing suite of instruments including VIRTIS-H (Bockelée-Morvan et al. 2016), MIRO (Biver et al. 2019), and Alice (Feldman et al. 2015; Keeney et al. 2017). The activity of water (Bieler et al. 2015b; Hansen et al. 2016; Kramer et al. 2017) and the major molecules (Biver et al. 2019; Bockelée-Morvan et al. 2016; Fougere et al. 2016; Läuter et al. 2019; Marshall et al. 2017) have been tracked by several instruments through large portions of the mission and their outgassing fluxes, both in absolute and relative numbers, exhibited a notable dependency on the heliocentric distance. The reported ratios were either derived at perihelion or represent relative abundances integrated over a large portion of the mission from pre- to post-perihelion. Table 2 lists some notable similarities as well as differences between *in situ* and remote sensing derived ratios, e.g. NH_3_ and OCS agree within a factor of 2 between ROSINA and VIRTIS-H/MIRO, respectively. All these data are also represented in Fig. 1.

**Table 2:** Measured coma abundances or abundance ranges by number (in % normalized to water) for a suite of volatile molecules from *in situ* ROSINA observations in May/June 2015 before perihelion (Rubin et al. (2019a) and references therein) and via remote sensing by MIRO, integrated over the 2 years Rosetta followed the comet (Biver et al. 2019), VIRTIS-H at perihelion (Bockelée-Morvan et al. 2016), and Alice from ∼2 au inbound to 2.5 au outbound (Keeney et al. 2017). Species observed by mass spectrometry may (also) be present in the form of different isomers (cf. Fig. 1)

Molecule	Abundances or abundance ranges relative to water [%]			
	ROSINA	VIRTIS-H	MIRO	Alice
H_2_O	100	100	100	100
CO_2_	4.7 ± 1.4	14 – 32		
CO	3.1 ± 0.9		0.6 ± 0.1	
O_2_	3.1 ± 1.1			11 – 68
CH_4_	0.34 ± 0.07	0.23 – 0.47		
C_2_H_6_	0.29 ± 0.06			
CH_3_OH	0.21 ± 0.06		1.5 ± 0.1	
H_2_CO	0.32 ± 0.10			
HCOOH	0.013 ± 0.008			
(CH_2_OH)_2_	0.011 ± 0.007			
CH_3_COOH	0.0034 ± 0.0020			
CH_3_CHO	0.047 ± 0.017			
CH_3_NO	0.0040 ± 0.0023			
NH_3_	0.67 ± 0.20		0.34 ± 0.01	
N_2_	0.089 ± 0.024			
HCN	0.14 ± 0.04			
HNCO	0.027 ± 0.016			
CH_3_CN	0.0059 ± 0.0034			
HC_3_N	0.00040 ± 0.00023			
H_2_S	1.10 ± 0.46			
OCS	$ 0.041_{-0.020}^{+0.082} $	0.12 – 0.18		
SO	$ 0.071_{-0.037}^{+0.142} $			
SO_2_	$ 0.127_{-0.064}^{+0.254} $			
CS_2_	$ 0.0057_{-0.0028}^{+0.0114} $			
S_2_	$ 0.0020_{-0.0010}^{+0.0040} $			
S	0.46 ± 0.36			
H_2_CS	$ 0.0027_{-0.0024}^{+0.0058} $			
CH_3_SH	$ 0.038_{-0.028}^{+0.079} $			
CH_3_CH_2_S CH_3_SCH_3_	$ 0.00058_{-0.00049}^{+0.00123} $			

In a different approach, Combi et al. (2020) integrated ROSINA-derived gas production rates between inbound and outbound equinoxes and obtained CO_2_/H_2_O = 7.4% and CO/H_2_O = 2.7%, which are both somewhat closer to the corresponding MIRO and VIRTIS-H values. Combi et al. (2020) also showed that the derived gas activities are particularly sensitive to the used models which turn column densities from remote-sensing observations or *in situ* densities from ROSINA into production rates. This is particularly important for observations in the far-ultraviolet (FUV), where energetic electrons are an important source for the dissociation of molecules (e.g. O_2_, CO, CO_2_) and formation of excited H, C, and O atoms (Feldman et al. (2015), cf. Mumma et al. (1971, 1972). Such analysis requires a detailed simulation of neutral gas and plasma, driven by the corresponding measurements from Rosetta including the Rosetta Plasma Consortium (Carr et al. 2007). It is important to note that models have improved significantly and the resulting relative as well as absolute production rates are converging. Nevertheless, the efforts in understanding the differences among the reported values from the different instruments are still ongoing. This is also interesting in the view of 67P/C-G’s next apparition in November 2021, which is much more favorable for a terrestrial observation campaign compared to summer 2015.

Figure 1 also reveals differences between 67P/C-G and other comets. This may either point at 67P/C-G exhibiting peculiar low abundances in species such as CS_2_ or, as mentioned above, may derive from the difficulty to remotely detect species with low (relative) abundances in distant comets.

The instrument on Rosetta dedicated to the composition of the refractory phase was the Cometary Secondary Ion Mass Analyzer (COSIMA; Kissel et al. 2007). It was found that the dust is rich in high molecular weight organic matter (Fray et al. 2016). Elemental ratios show carbon-rich dust (Bardyn et al. 2017) with a C/Si ratio comparable to solar relative abundances and earlier measurements at 1P/Halley by the PUMA-1 instrument onboard Vega 1 (Jessberger et al. 1988). The authors concluded that by weight the dust is composed of nearly equal amounts of organic matter and anhydrous mineral phases, with up to 90% porosity (Langevin et al. 2017). The N/C atomic ratio of the organic matter in 67P/C-G matches that of insoluble organic matter (IOM) chemically extracted from primitive meteorites (Fray et al. 2017), but its higher H/C ratio compared to IOM suggests a more unsaturated state in 67P/C-G (Isnard et al. 2019).

The elemental composition of dust of 67P/C-G is broadly compatible with chondritic composition, but also exhibits notable differences, as Mg, Ca, and Fe are significantly depleted w.r.t. the chondritic composition (Bardyn et al. 2017). When compared to different types of meteorites, 67P/C-G dust bears more similarities to carbonaceous chondrite composition, than to other types of meteorites, but there is no perfect match (Stenzel et al. 2017).

**Fig. 2:**
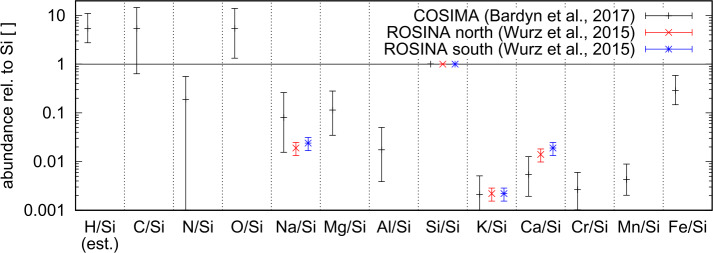
Abundances of the elements in the refractory phase relative to silicon measured by COSIMA (black; Bardyn et al. 2017) and ROSINA (Wurz et al. 2015) at comet 67P/C-G. The COSIMA measurement represents an average of the analyzed dust grains and the ROSINA observations are the individually averaged sputter-signals above the at the time more active northern summer hemisphere (red) and the lesser active southern winter hemisphere (blue). The H/Si ratio is an estimate from Bardyn et al. (2017)

ROSINA DFMS, as mentioned earlier, was dedicated to measure the neutral gas species in the coma. Early in the mission, DFMS measured several refractory elements in the coma (Wurz et al. 2015). It was established, that at 67P/C-G’s low cometary activity beyond 3 au, the solar wind reached at least parts of the surface of the nucleus and subsequent sputtering released refractory elements into the coma. DFMS detected atomic Na, Si, K, and Ca, however, none of them were in molecular form. From these measurements their relative abundances have been derived. Figure 2 shows a comparison between COSIMA (Bardyn et al. 2017) and ROSINA (Wurz et al. 2015) derived ratios normalized to Si. With ROSINA, the small sputtered contribution of H, C, and O cannot be distinguished from the fragments of the abundant volatile organic and inorganic molecules formed by electron-impact inside the ion source of DFMS. Therefore, a meaningful comparison between these elements is not possible. However, the relative abundances of the measured refractory elements overlap at the 1-$\sigma $ level of the corresponding COSIMA observations. ROSINA measurements show a high relative abundance of Si and therefore support the conclusion by Bardyn et al. (2017) that the mineral phase in 67P/C-G’s dust is predominantly composed of anhydrous silicates.

## Conditions at the Location of Formation of Comets

### Comet Formation

The classic view has the solar system forming as a consequence of the gravitational collapse of an interstellar cloud, likely a giant molecular cloud. Such star-forming regions seen today often display multiple “hot cores” that are collapsing into individual stars and their planetary systems. The solar system’s birth cloud likely featured many such cores, with one leading eventually to the Sun and planets of our evolved system. Most of the core mass wound up in the Sun but because the core was slowly rotating, a small fraction formed a protoplanetary disk and its surrounding gravitationally attached envelope of dust and gas (the ensemble being called the “Solar nebula”). The protoplanetary disk defined the equatorial plane of the nebula, with disk material and its gaseous environs rotating around the central star and flaring in height at large heliocentric distances. This disk was composed of gas plus sub-micron and larger particles of ice and dust (though the ice sublimated away close to the Sun or, upon arrival at the surface of the accretion disk, the infalling material may have crossed a shock and been evaporated (Chick and Cassen 1997)). This material slowly coalesced through a process known as hierarchical accretion, particles running into one another and sticking (Weidenschilling 1977, 1997). Thus, particles grew in size.

Numerical simulations show that micron-sized particles can grow through hierarchical accretion to centimeter size but as they approach meter-size, their inter-particle collisions increase in velocity and change from accretional to erosional (Blum 2010, 2018). Compaction of the growing particles during collisions also increases their strength and they begin to bounce off one another.

There are two hypotheses proposed to surmount this growth barrier. The first is that hierarchical accretion somehow continues to larger sizes creating kilometer-sized bodies. The presence of water ice may increase the growth rate past the 1-meter barrier due to the stickiness of water ice (Ros and Johansen 2013). These are the forerunners of the comet nuclei we see today (Davidsson et al. 2016). The second hypothesis is that when particles grow to centimeter sizes they are brought together in streaming (hydrodynamic) instabilities in the solar nebula which then gravitationally collapse into macroscopic bodies on the order of 50 to 100 km in diameter (Youdin and Goodman 2005). Subsequent collisions create both smaller and larger bodies, all of which can be characterized as icy planetesimals (future comets), though the smaller ones are far more numerous.

Note that the distinguishing feature of comets is that they contain substantial amounts of volatiles, primarily water ice, aside from refractory material (Choukroun et al. 2020). Therefore, comets must have formed in colder regions of the solar nebula where water ice could be stable. In the current solar system, the “snowline” is found around 3-5 au (Min et al. 2011). But in the solar nebula it may have been closer to the Sun. In a disk solely illuminated by the star, the snowline would be inward of 1 au, because of the low optical depth (Chiang and Youdin 2010; Sasselov and Lecar 2000). However, the disk can be heated by viscous dissipation, so that the location of the snowline depends on the stellar accretion rate (Bitsch et al. 2015; Oka et al. 2011), with a snowline up to 4-5 au when the star accretes at 10^−7^ M_Sun_/year (early disk). However, some of the more volatile species found in comets, e.g., N_2_, CO, etc. suggest formation temperatures on the order of 30 K or less, and thus require formation and capture considerably farther out.

Regardless of which formation mechanism is correct, the comet nuclei likely continued to evolve collisionally before their dynamical dispersion to the Kuiper belt and Scattered disk, and/or to the Oort cloud (Morbidelli and Rickman 2015). Subtle physical processes, such as heating by nearby supernovae and cosmic rays will also modestly process the near-surface layers of the nuclei. These topics and related subjects are discussed in detail in the paper by Weissman et al. (2020) and later in Sect. 5.

### On the Refractory (Dust) Phase of 67P/C-G and Other Comets

Cometary dust composition data are available for 67P/C-G (Bardyn et al. 2017; Fray et al. 2016, 2017; Isnard et al. 2019; Wurz et al. 2015) and a few other comets: 1P/Halley (Giotto and Vega), (e.g. Jessberger et al. 1988; Kissel et al. 1986a,b), 81P/Wild 2 (e.g. Brownlee 2014 and references therein), 9P/Temple 1 (Lisse et al. 2006), and C/1995 O1 (Hale-Bopp) (e.g. Crovisier et al. 1997). A detailed review can be found in Levasseur-Regourd et al. (2018).

The minerals found in these comets show variation in their nature from one comet to another, but mostly consist of crystalline minerals (refractory oxides and silicates, olivine, pyroxenes, metal, sulfides and accessory minerals) as well as amorphous silicates. The crystalline minerals identified in the comets were formed at high temperature close to the early Sun and subsequently transported to the external regions of the protoplanetary disk, in the comet forming region (e.g. Bockelée–Morvan et al. 2002; Ciesla 2007; Shu et al. 1997; Vinković 2009).

The most detailed mineralogical characterization was possible for 81P/Wild 2 samples brought back by the Stardust mission. These samples contain refractory minerals (Ca-Al-rich inclusions) as well as chondrule fragments and crystalline silicates similar to those identified in primitive meteorites (e.g. Brownlee et al. 2006; Nakamura et al. 2008; Zolensky et al. 2006). The olivines from the Stardust sample show a very large variation range of their major and minor element concentrations, relating them to olivines found in primitive (carbonaceous) chondrites, but also to olivines from ordinary chondrites (Frank et al. 2014). These very diverse olivine compositions and the lack of ^26^Al in refractory minerals in Wild 2 samples (e.g. Nakashima et al. 2015) could mean that comet 81P/Wild2 incorporated minerals that formed late (without ^26^Al) and that had been already processed (e.g. in an ordinary chondrite parent body). These minerals were therefore most likely accreted after the formation of the icy cometary body.

Hints for traces of aqueous alteration were found in comet Wild 2 minerals (e.g. Berger et al. 2011; Mikouchi et al. 2007; Hicks et al. 2017; Hanner and Zolensky 2010). Spitzer spectra of 9P/Tempel 1 acquired during the Deep Impact mission could contain a contribution from a few percent of hydrated silicates, although this is debated (Lisse et al. 2006).

Chondritic porous interplanetary dust particles (IDPs), that are probably of cometary origin, are mostly anhydrous, but they can contain trace amounts of hydrated minerals (e.g., Bradley 2003; Brownlee 2014). Ultracarbonaceous Antarctic Micrometeorites (UCAMMs) that are probably of cometary origin could also have experienced minimal aqueous alteration (Guérin et al. 2020; Yabuta et al. 2017). Whether these small amounts of aqueous alteration products found in comets were produced *in situ* or accreted from previous episodes is still debated (Suttle et al. 2020).

### On the Ice Phase and Formation Temperature of 67P/C-G

Rubin et al. (2015a) reported N_2_/CO to be depleted in comet 67P/C-G by a factor of $25.4 \pm 8.9$ relative to protosolar N and C abundances. One interpretation is the slightly different trapping efficiency of N_2_ with respect to CO in amorphous ice. Hence N_2_/CO is temperature-dependent and the measured ratio of $5.7\cdot 10^{-3}$ at the comet, compared to the protosolar nebula value $0.145 \pm 0.048$ (Lodders et al. 2009) and assuming all N was in N_2_ and C was in CO, led to a formation temperature below ∼30 K for 67P/C-G. Computations based on clathrate hydrates for N_2_/CO and Ar/CO reveal a somewhat higher formation temperature, though still below 50 K (Mousis et al. 2012, 2016a). However, this N_2_/CO ratio was obtained at a heliocentric distance beyond 3 au. Relative abundances of various volatiles including the major species H_2_O, CO, CO_2_, and O_2_ changed over the course of comet 67P/C-G’s eccentric orbit around the Sun (Biver et al. 2019; Bockelée-Morvan et al. 2016; Fougere et al. 2016; Keeney et al. 2017). In particular the relative abundance of CO with respect to water decreased due to the sharply increasing water production rate closer to the sun (Läuter et al. 2019). Furthermore, the relative abundance of other volatile molecules also increased towards perihelion, even for species of similar volatility such as N_2_. A suitable period to derive bulk abundances has been identified to be May 2015, a few months before perihelion passage (Calmonte et al. 2016). If we combine the reported N_2_/H$_{2}\text{O} = 8.9 \cdot 10^{-4}$ ratio from Rubin et al. (2018) and CO/H$_{2}\text{O} = 0.031$ ratio estimated from Rubin et al. (2019a) for the same period, a roughly 5 times larger N_2_/CO ratio and hence a lower depletion rate of N_2_ with respect to CO is obtained. In the case of amorphous ice, such a ratio would move the formation temperature of 67P/C-G towards even lower values, possibly into the low 20 K range (Bar-Nun et al. 2007). However, such a scenario would also lead to an even higher Ar/CO ratio, which is not observed and is at odds with the absence of the noble gas neon in the coma of comet 67P/C-G (Bar-Nun et al. 2012; Rubin et al. 2018). What remains to be understood, however, is the impact of other ices aside from H_2_O. For instance the addition of CO_2_, the second most abundant species in the coma of comet 67P/C-G (Fougere et al. 2016; Läuter et al. 2019), has been shown to increase trapping efficiencies of N_2_ and Ar (Greenberg et al. 2017). Late in the Rosetta mission, at heliocentric distances >3 au, when the noble gases Xe, Kr, and Ar were observed, CO_2_ was the dominant volatile in the coma (Läuter et al. 2019). The jury is still out on whether highly volatile species are predominantly trapped in CO_2_ as opposed to H_2_O in the case of amorphous ices.

However, this discussion must be revisited in the context of the recent discovery of abundant ammonium salts in 67P, that suggest the ‘missing’ N in comets may be hidden in them (Altwegg et al. 2020; Poch et al. 2020). These semi-volatile salts can form in the interstellar medium at temperatures below 15 K and then remain stable until warmed to much higher temperatures (see later discussion). They can be carried into pre-cometary ices and survive until released and sublimated in the inner solar system, releasing NH_3_, HCN, organic acids, and other products into the cometary coma (Mumma et al. 2019).

**Fig. 3:**
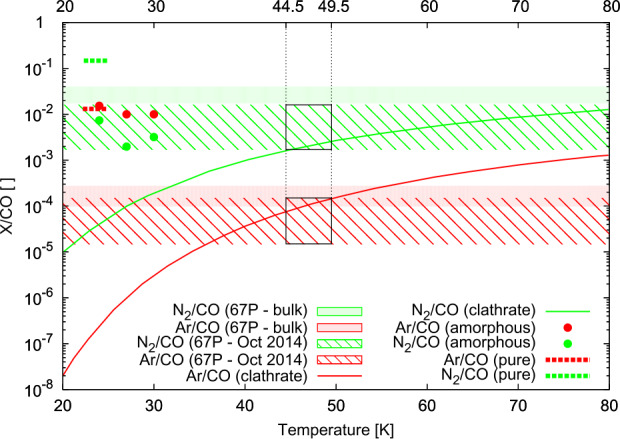
N_2_/CO (green) and Ar/CO (red) ratios measured at comet 67P/C-G and obtained from water ice experiments and statistical calculations of H_2_O guest clathrate adapted from Mousis et al. (2016a). The measured ranges of the ratios differ by approximately a factor 10 between October 2014 when Rosetta was beyond 3 au (hatched area, data from Balsiger et al. 2015 and Rubin et al. 2015a) and the comet’s near-perihelion production rate ratios (solid area, numbers derived from Rubin et al. 2018 and Rubin et al. 2019a). Relative abundances of pure ice condensation are shown by the dashed lines at temperatures <25 K (Mousis et al. 2010) and trapping experiments in amorphous ices obtained at 24 K, 27 K, and 30 K (Bar-Nun et al. 2007) are represented by dots. Relative abundances from statistical thermodynamics modeling of clathrate hydrates along the equilibrium temperature curves are plotted in solid lines. The temperature range from 44.5 – 49.5 K simultaneously overlapping N_2_/CO and Ar/CO ratios modeled and the October 2014 Rosetta measurements are indicated by the black boxes

Scenarios of evaporation of amorphous ices (Chick and Cassen 1997; Mousis 2000) and recondensation in the protosolar nebula (Kouchi et al. 1994) have also been discussed in the literature. Figure 3 shows the measured N_2_/CO and Ar/CO ratios at comet 67P/C-G for heliocentric distances >3 au and derived production rate ratios near perihelion in comparison to ratios derived from relative trapping as pure ices, in amorphous ices, and in clathrate ices (adopted from Mousis et al. 2016a). While from the early measurements an overlapping formation temperature reproducing observed Ar/CO and N_2_/CO could be derived in the case of clathrate hydrates, this is no more possible for the measured bulk abundances. Further ice trapping experiments and statistical thermodynamics models (Lunine and Stevenson 1985) of N_2_, noble gases, and CO trapping in comet-style ices, containing not only H_2_O but also CO_2_, still have to be carried out. This mix of ices is especially important given that the noble gases were better correlated with CO_2_ than with H_2_O. Nevertheless, these results show that the ices of a comet cannot be represented by one single phase of water ice. This is consistent with the recent scenario proposed by Mousis et al. (2018b), where the ices are inherited from the presolar cloud but then undergo a phase transition from amorphous to crystalline/clathrate ices including retrapping of the more volatile species, with a few exceptions that will be discussed later in Sect. 4.2.

Most of these calculations were based on solar abundances (Lodders et al. 2009). Analysis including carbon monoxide and molecular nitrogen was performed on the assumption that C being predominantly incorporated in CO and N in N_2_ (Fegley and Prinn 1989) and hence the relative amounts of these two molecules in the interstellar medium (ISM) and the protosolar nebula (PSN), respectively, were derived from the solar N/C ratio.

To complicate matters further, later evolutionary processes have to be considered for the comet’s journey from the Scattered disk to the inner planetary system, i.e. the loss of preferentially highly volatile species by heating through collisional interactions among cometesimals or gradual warming of the nucleus during the intermediate Centaur stage. These topics will be discussed in Sect. 5.

## Origin of Cometary Material

### Isotopes

The isotopes found in cometary refractories and volatiles contain information on the origin and the processing of the material. In their work, Bockelée-Morvan et al. (2015) reviewed the suite of D/H, ^14^N/^15^N, ^16^O/^18^O, ^12^C/^13^C, and ^32^S/^34^S ratios measured in cometary material and discussed their role as tracers for the physical chemical conditions responsible for the observed isotopic fractionation. In the following we will review some of these measurements with a focus on comet 67P/C-G and discuss their relevance with respect to their formation history. A complementary comparison of Rosetta-derived isotopic compositions with solar system materials and the ISM can be found in Hoppe et al. (2018) and Levasseur-Regourd et al. (2018).

#### The Variation in D/H

Generally, due to the high relative mass difference between deuterium and hydrogen, the D/H ratio is most prone to fractionation effects. A key measurement is thus the D/H ratio in the cometary H_2_O molecule. It has been used to infer potential sources for the water on Earth and other objects in the solar system. It has been recognized that the degree of fractionation depends on the environmental conditions under which the ices formed, for example, on grain surfaces in molecular clouds. Grain surface reactions at low temperatures (∼10 K) can enhance the deuterium fractionation considerably (Brown and Millar 1989a,b; Charnley et al. 1997). In particular, it has been predicted that the D/H ratio increases with heliocentric distance of the formation location of the ices (Aikawa and Herbst 2001). The cause for this effect is the difference in the zero-point energy between D- and H-bearing molecules. Thus in the cold environment D and H substitution reaction rates are not equal, favoring the substitution of H by D in a molecule (Ceccarelli et al. 2014).

The D/H ratio in water has been observed in more than a dozen comets, from both the Oort cloud and the Kuiper belt (for recent summaries see Paganini et al. 2017; Lis et al. 2019). The variation in the D/H ratio in water can vary by a factor of a few but most comets show deuterium enrichment with respect to the Earth, i.e. the Vienna Standard Mean Ocean Water (VSMOW) of $\text{D}/\text{H}= (1.558 \pm 0.001) \cdot 10^{-4}$, which is already much higher than the protosolar D/H ratio in H_2_ of $2 \cdot 10^{-5}$ (Lodders et al. 2010). The early measurements of D/H in H_2_O were all performed on Oort cloud comets and showed enrichment compared to VSMOW. After the observation of a terrestrial D/H ratio in comet 103P/Hartley 2, however, it was hypothesized that Jupiter-family comets (JFCs) may have contributed sizeable amounts of water to the Earth (Hartogh et al. 2011). JFC 67P/C-G, on the other hand, yielded a much higher D/H ratio, more than 3.5 times the ratio on Earth (Altwegg et al. 2015). The two consequences are that the origin of terrestrial water is again unresolved and that dynamical families of comets cannot simply be distinguished by their D/H ratios. The D/H ratio in cometary water could instead be the result of a mixture of presolar, high D/H water with isotopically lighter water from the inner solar system. In summary, it is thus hypothesized that the measured D/H ratio rather reflects the formation location of the comet before being expelled through the migration of the giant planets (Gomes et al. 2005). A more recent study by Lis et al. (2019), after the observation of a D/H ratio in comet 46P/Wirtanen compatible with VSMOW, suggested a correlation of the D/H ratio with the size and activity of a comet. Hyperactive comets, such as 103P/Hartley 2 and 46P/Wirtanen may therefore contain more ices processed in the inner solar system, possibly locked-up in icy grains sublimating only after their release into the coma. A recent study by Schroeder et al. (2019) also showed that, within error, the water above both lobes shares the same D/H ratio. This hints at a formation of both lobes of the comet in the same region of the protoplanetary disk before the final collisional merger occurred.

Still, the question whether parts of the ice are even inherited from the presolar cloud is difficult to answer from the ratio of HDO/H_2_O alone. Furuya et al. (2016) showed that water can retain its high HDO/H_2_O ratio even if it is reprocessed in the protostellar disk. Nevertheless, the D/H ratios in comets are often lower than ratios observed near young stellar objects. Further complication arose from observations of much higher D/H ratios in doubly versus singly deuterated water vapor of [D_2_O/HDO]/[HDO/H_2_O] ≅ 7 around low-mass protostar NGC1333 IRAS2A (Coutens et al. 2014). Furuya et al. (2016) therefore presented a model where the deuteration of water occurs in a two-step process: in stage 1, H_2_O forms on the surface of grains with a D/H ratio reflecting the surrounding environment; in stage 2, as the molecular cloud cools down, water production is reduced but deuteration becomes much more efficient due to the involvement of non-equilibrium chemistry at low temperatures. The model follows the earlier work by Dartois et al. (2003), based on observations of even higher D/H ratios in molecules freezing out at lower temperatures compared to water, e.g. in NH_3_ (Lis et al. 2002; Roueff et al. 2000; Shah and Wootten 2001; Van der Tak et al. 2002), CH_3_OH (Parise et al. 2006), and H_2_S (Hatchell et al. 1999). In stage 2, where most of HDO and D_2_O is formed, the deposition of CO also leads to the formation of H_2_CO and CH_3_OH which are thus subject to higher D/H fractionation. Thus, Furuya et al. (2016) predicted D/H ratios from CH_3_OD/CH_3_OH to be similar to D_2_O/HDO but larger than from HDO/H_2_O, although the situation becomes more complicated once additional substitution and abstraction reactions are involved, e.g. in the case of CH_2_DOH/CH_3_OH.

A similar picture has been obtained for comet 67P/C-G when comparing the two different D/H ratios derived in water. HDO/H_2_O corresponds to $2\cdot \text{D}$/H as D in HDO can sit on either of the two positions of hydrogen in the water molecule and, likewise, D_2_O/HDO corresponds to $1/2$ ⋅ D/H. From statistics one would therefore expect $1/4$ for D_2_O/HDO relative to HDO/H_2_O in an equilibrated system, where D/H matches in both ratios. Altwegg et al. (2017a), however, reported a D_2_O/HDO relative to HDO/H_2_O ratio of 17. Furthermore, the same authors found HDS/H_2_S = $(1.2 \pm 0.3) \cdot 10^{-3}$, a ratio which overlaps that of HDO/H_2_O = $(1.05 \pm 0.14) \cdot 10^{-3}$ within 1-$\sigma $ uncertainties, also in line with Furuya et al. (2016) where H_2_S is formed in the colder stage 2. Around the cold star-forming core IRAS 16293 an even higher ratio of HDS/H_2_S = 0.1 has been derived (van Dishoeck et al. 1995). High D/H ratios have also been observed in other species such as HCN in comet Hale-Bopp with D/H = $(2.3 \pm 0.4) \cdot 10^{-3}$ (Meier et al. 1998a) and D/H = $(2.3 \pm 0.6) \cdot 10^{-3}$ (Crovisier et al. 2004). However, these ratios are still small compared to the ISM observations, which yield D/H in the range of $(0.4 - 7) \cdot 10^{-2}$ (Jørgensen et al. 2004; Roberts et al. 2002) and thus Bockelée-Morvan et al. (2015) argued for some degree of reprocessing in the solar nebula before incorporation into the comet.

**Fig. 4:**
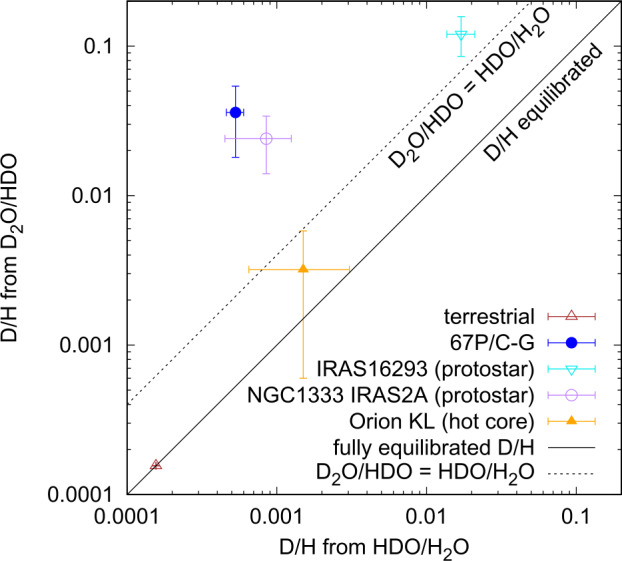
D/H ratio obtained from HDO/H_2_O versus D/H obtained from D_2_O/HDO in comet 67P/C-G (Altwegg et al. 2017a), the two protostars IRAS 16293 (Coutens et al. 2012; Le Roy et al. 2015) and NGC1333 IRAS 2A (Coutens et al. 2014; Rubin et al. 2019a), and the Orion KL hot core (Dello Russo et al. 2016; Neill et al. 2013) compared to the terrestrial value (Haynes 2013). The solid black line corresponds to equal D/H ratios in both HDO/H_2_O and D_2_O/HDO. The dashed line represents where HDO/H_2_O is equal to D_2_O/HDO proposed by Furuya et al. (2016) to distinguish unprocessed from processed ices. Error bars are 1-$\sigma$

Figure 4 shows the D/H ratios in singly and doubly deuterated water observed in several objects including the Earth, comet 67P/C-G, and in star forming regions. The non-equilibrated D/H ratio in 67P/C-G is similar to the observations around protostars IRAS 16293 and NGC1333 IRAS2A, while the warmer environment of the Orion KL hot core leads to a similar D/H ratio in both D_2_O/HDO and HDO/H_2_O. In liquid water, hydrogen and deuterium atoms are rapidly exchanged, which leads to an equilibrated D/H ratio in the terrestrial oceans. Nevertheless, in the case of equilibration, a high D/H in D_2_O/HDO only marginally affects the original HDO/H_2_O ratio due to the low relative abundance of D_2_O.

In their work, Furuya et al. (2016) concluded that the ratio of [D_2_O/HDO]/[HDO/H_2_O] reveals much more about the formation of the ice, whether it is of presolar origin or has been reformed or reprocessed in the protosolar nebula: laboratory experiments show that H-D exchange in hydrogen-bonded molecules in mixed amorphous ices occurs efficiently on timescales of the order of 10^4^ years at temperatures ≳70 K, well below the crystalline ice transition temperature (Faure et al. 2015; Lamberts et al. 2015; Mousis et al. 2016a; Ratajczak et al. 2009). On the other hand, given the two-stage setup of the model the highly deuterated water molecules HDO and D_2_O from the outer layers are not intimately mixed with the bulk H_2_O which reduces the efficiency of the H-D exchange and equilibration. Nevertheless, H-D isotope exchange would also affect other species and lead to the reprocessing of the DCN/HCN ratio.

In summary the model by Furuya et al. (2016) predicts [D_2_O/HDO]/[HDO/H_2_O] ratios that are generally ≫1 around solar-type protostars while in stellar nebulae where ice is reprocessed the obtained ratios are ≲1 (cf. Altwegg et al. 2017a and references therein). The inheritance of two different D/H ratios in singly and doubly deuterated water therefore indicates that at least parts of the ices in comet 67P/C-G originated in the presolar cloud and survived the incorporation into the comet. Furthermore, different D/H ratios in singly versus doubly deuterated water emphasizes that isotopic equilibration in the ices of a comet are limited after its formation. To which extent D-H exchange can be inhibited in the alternative scenario of warmed-up ice agglomeration by Mousis et al. (2018b) remains to be investigated.

As Bockelée-Morvan et al. (2015) pointed out, the HDO/H_2_O ratio in comets is typically lower compared to the ISM. Possible explanations discussed are ion-molecule reactions in the outer solar nebula (Aikawa and Herbst 1999; Meier et al. 1998b) or the mixing of D-rich ices from the outer solar system with processed material transported to the formation location of the comet (Mousis 2000) as water vapor readily exchanges D and H atoms with the H_2_, the main hydrogen reservoir (Geiss and Gloeckler 1998). It is not entirely clear, however, how a D/H ratio different in the two ratios HDO/H_2_O and HDO/D_2_O can be maintained. Another consideration is thus a consequence of the 2-stage model: HDO and D_2_O are associated to the layer of species more volatile than water. If co-desorption together with highly volatile molecules, such as CO, occurs, the D/H ratio exhibits a temperature-dependence with high D/H ratios below the water desorption temperature of >140 K (cf. Kouchi and Yamamoto 1995). This is in line with measurements in the ISM where cold gas exhibits much higher D/H ratios compared to warm gas (cf. Persson et al. 2014 and references therein). At the comet, unfortunately, observing such an effect, where outgassing occurs from a most likely thermally altered subsurface layer and locations of different temperatures, would be very difficult.

Also in the refractory material returned by the Stardust mission moderately elevated D/H ratios have been observed (McKeegan et al. 2006). The absence of hydrated minerals, however, led Bockelée-Morvan et al. (2015) to the conclusion that the measured D/H ratios in the Stardust samples are not representative of the D/H ratio in the ices of 81P/Wild 2. The D/H ratio in dust particles from 67P/C-G seems to be about ten times the terrestrial reference (Paquette et al. 2018a). However, this probably refers to the D/H ratio of the organic matter, as no hydrated minerals were identified on 67P/C-G’s surface by the VIRTIS instrument (Quirico et al. 2016) and there was no evidence for the presence of hydrated silicates in the dust collected in the coma and analyzed by COSIMA (Bardyn et al. 2017).

#### Nitrogen Isotopes

The nitrogen isotope ratio shows remarkable variations across the solar system. Starting from the variation of the ^14^N/^15^N ratios observed in the interstellar medium to the more or less uniform value in the molecules observed in comets, to the Sun and the planets makes this an important object to study the provenance of the material in the solar system.

Nitrogen isotopes in the three molecules HCN, CN, and NH_2_, have been measured in numerous comets (Bockelée-Morvan et al. 2008; Manfroid et al. 2009; Rousselot et al. 2014; Shinnaka et al. 2014) including C/1995 O1 (Hale-Bopp), 73P/Schwassmann-Wachmann 3, C/2012 F6 (Lemmon), and C/2012 S1 (ISON) among others. For 67P/C-G the nitrogen isotope ratios are still being analyzed. While nitrogen isotopic ratios in comets range from ^14^N/^15^N = 90 – 220 (cf. Bockelée-Morvan et al. 2015 and Manfroid et al. 2009) the average ratio derived by Hily-Blant et al. (2017) of ^14^N/^15^N = 144 ± 3 is consistent through the three cometary molecules listed above.

However, this ratio is distinctly lower (i.e., ^15^N enriched) than the solar wind and protosolar nebula values of ^14^N/^15^N = 441 ± 6 and ^14^N/^15^N = 459 ± 4, obtained and derived from the Genesis mission (Marty et al. 2011), which are consistent with the ratios obtained in Jupiter (Fletcher et al. 2014; Fouchet et al. 2000; Owen et al. 2001). Also the terrestrial ratio of ^14^N/^15^N = 273 ± 1 (Nier 1950) is significantly higher compared to cometary material. The degradation of ammonium salts can release all three species (HCN, CN, NH_2_) in the coma, complicating the interpretation since isotopic ratios in stored ices and salts may differ.

The refractory samples returned by the Stardust mission to comet 81P/Wild 2 showed a large variation in individual grains, ranging from the protosolar value for a few grains which also showed enrichment in ^13^C compared to terrestrial abundances all the way to a few ^15^N hotspots, i.e. grains with ^14^N/^15^N ratios similar to cometary volatiles. The bulk composition was clustering somewhere between terrestrial and elevated ^15^N, i.e. ^14^N/^15^N = $180 - 270$ (McKeegan et al. 2006).

Unlike the volatile species in comets, the nitrogen isotope ratios in the interstellar medium around protostars differ significantly among the objects and the investigated molecules (Füri and Marty 2015). Evidence for multiple reservoirs of nitrogen have been found (Hily-Blant et al. 2017; Rodgers and Charnley 2008). Hily-Blant et al. (2013) presented a chemical network of gas-phase reactions where the two different reservoirs of molecular and atomic nitrogen have different isotope ratios (Furuya et al. 2018). From these two reservoirs the amines and nitriles form in separate pathways which thus leads to the differences of the ^14^N/^15^N ratios in NH, CN, HCN^(+)^, HNC versus N_2_H^+^, NH_x_^(+)^ observed in the ISM (Füri and Marty 2015) and references therein), while in comets the ^14^N/^15^N ratios in HCN, CN, and NH_2_ are quite uniform. Furuya et al. (2018) thus argue that the differences in N-bearing species between comets and interstellar ices indicates either a primordial variation or ice processing in the solar nebula (Furuya and Aikawa 2014; Lyons et al. 2009). Release from ammonium salts may further complicate this picture.

Two major fractionation mechanisms have been discussed in the literature. Chemical fractionation occurs when the rates for ^15^N-^14^N exchange reactions in nitrogen-bearing species deviate at low temperatures (∼10 K), i.e. when the activation energy barriers quench the ^14^N enrichment (Hily-Blant et al. 2013; Rodgers and Charnley 2008). Self-shielding, on the other hand, affects the dissociation of molecules in dense clouds as the attenuation of the incoming radiation is isotope-selective. Therefore, the ^14^N/^15^N ratio in molecules such as N_2_ depends on the location within the cloud (Heays et al. 2014). The importance of selective dissociation of N_2_ is supported in a recent work by Hily-Blant et al. (2019), who presented evidence for a gradient in the HCN/HC^15^N ratio as a function of radial distance in the protoplanetary disk of 8 Myr young T Tauri star TW Hya. Around 20 au, a typical region for the formation of comets, the resulting ratio is HCN/HC^15^N = 121 ± 11, similar to the one observed in the solar system comets, whereas farther out the ratio is close to the local interstellar medium.

#### Carbon and Oxygen Isotopes

Hässig et al. (2017) presented a collection of measured oxygen and carbon isotopes in volatile species at various comets. Among the comets with known ^16^O/^18^O ratio in H_2_O and CO_2_ are 1P/Halley (Balsiger et al. 1995; Eberhardt et al. 1995), 153P/Ikeya–Zhang, C/2002 T7 (LINEAR), C/2001 Q4 (NEAT), C/2004 Q2 (Machholz) (Biver et al. 2007; Hutsemékers et al. 2008), C/2012 F6 (Lemmon, Bockelée-Morvan et al. (2012)), and 67P/C-G (Schroeder et al. (2018), revised from Altwegg et al. (2015)). A non-exhaustive list of comets with measured ^12^C/^13^C ratios in C_2_, CN, HCN C_2_H_x_, CO, and CO_2_ contains 1P/Halley (Kleine et al. 1995), C/1995 O1 (Hale-Bopp, Jewitt et al. (1997)), and 67P/C-G (Hässig et al. 2017; Rubin et al. 2017).

Generally, the deviations of ^16^O/^18^O and ^12^C/^13^C measured at comets with respect to solar are smaller compared to D/H and ^14^N/^15^N (cf. Sects. 4.1.1 and 4.1.2), and often the associated accuracies do not assert a clear distinction from the solar reference ratios of ^12^C/^13^C = 98 ± 2 (Hashizume et al. 2004) and ^16^O/^18^O = 530 (McKeegan et al. 2011). Thus carbon isotopes in most comets were shown to be consistent with the solar reference, with the notable exceptions of C_2_ in comets West 1976 VI (Lambert and Danks 1983) and CO_2_ in 67P/C-G (Hässig et al. 2017), and a similar picture holds for the oxygen isotopes. In 67P/C-G, Hässig et al. (2017) and Schroeder et al. (2018) derived a depletion of ^16^O with respect to ^18^O in CO_2_ and ^16^O with respect to both ^17^O and ^18^O in H_2_O, respectively. Elevated ^13^C and ^18^O abundances can be the result of isotope-selective photodissociation due to self-shielding as discussed in Sect. 4.1.2. As Bockelée-Morvan et al. (2015) pointed out, this process was studied to understand the oxygen isotope ratios in primitive meteoritic matter (Lee et al. 2008), as the fractionation due to ion-molecule reactions proved to be negligible (Langer et al. 1984). In case of the volatiles, the measured effect in the oxygen isotopes of water is consistent with the predictions for primordial water from self-shielding models (Sakamoto et al. 2007; Yurimoto and Kuramoto 2004). This further strengthens the argument that at least some of the water in comet 67P/C-G is inherited from the presolar cloud.

The oxygen isotopic composition of cometary dust is usually compatible with that observed in carbonaceous chondrites, spanning a range from the solar value to the terrestrial value (e.g. Bockelée-Morvan et al. (2015), Levasseur-Regourd et al. (2018), Paquette et al. (2018b)). The carbon isotopic compositions of cometary dust show only small variations with regard to the terrestrial value (e.g. Levasseur-Regourd et al. (2018)).

#### Noble Gas Isotopes

Noble gases have been detected in the coma of comet 67P/C-G. The measured isotope ratios of argon (Balsiger et al. 2015) and krypton (Rubin et al. 2018) have been shown to be consistent with solar isotope ratios. The xenon isotopes, on the other hand, show remarkable differences compared to the solar system bulk (Marty et al. 2017). In particular the heavy isotopes, ^134^Xe and ^136^Xe, exhibit a strong depletion. As such the Xe-isotopes resemble the previously postulated primordial U-Xe (Pepin 2000), required to explain the xenon isotopic composition in the Earth’s atmosphere. A $22 \pm 5$% contribution of cometary xenon was derived by Marty et al. (2017). The authors suggested that the xenon in 67P/C-G can be reproduced by a mixture of different nucleosynthetic processes. However, the same approach cannot easily be applied to krypton. Thus, similar to the case of meteoritic Kr and Xe isotopes (Gilmour 2010), Rubin et al. (2018) showed that a mix of two nucleosynthetic end-members, i.e. an exotic s-process krypton to a “normal” component resembling solar composition, can indeed reproduce the observed krypton and xenon isotopic ratios at comet 67P/C-G. However, this also implies an excess of an exotic s-process component at the location of comet formation and thus a non-homogenized protosolar disk.

#### Sulfur Isotopes

Sulfur is an abundant species found in many comets (Altwegg 1995; Biver et al. 2016; Calmonte et al. 2016; Crovisier et al. 2004; Jewitt et al. 1997). Correspondingly, the isotopes of sulfur have also been observed in the volatiles of several comets, from *in situ* atomic S at comet 1P/Halley (Altwegg 1995) by the Giotto mission and in H_2_S, CS, and OCS at comet 67P/C-G (Calmonte et al. 2017), as well as by remote sensing observations of H_2_S and CS in Hale-Bopp by Jewitt et al. (1997) and Crovisier et al. (2004), respectively, and CS in both comets C/2013 R1 (Lovejoy) and C/2012 F6 (Lemmon) (Biver et al. 2016). Furthermore, sulfur isotopes have also been measured in the refractory material of comet Wild 2 returned by the Stardust mission (Heck et al. 2012) and at 67P/C-G by the COSIMA dust mass spectrometer (Paquette et al. 2017). The reference standard is obtained from Vienna-Canyon Diablo Troilite (V-CDT) and amounts to ^32^S/^33^S = 126.948 and ^32^S/^34^S = 22.6436 (Ding et al. 2001). The relative sulfur abundances have never been measured in the Sun or the solar wind and thus the reference is based on meteoritic material.

**Fig. 5:**
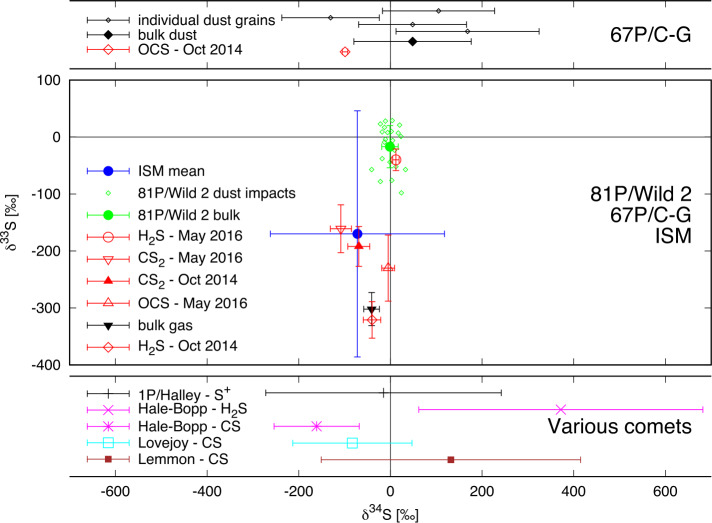
Sulfur isotope measurements in comets in $\delta $-notation. Bottom panel: $\delta ^{34}$S isotopic deviation at various comets with respect to V-CDT including atomic S in 1P/Halley: Altwegg (1995), H_2_S and CS in Hale-Bopp: Jewitt et al. (1997) and Crovisier et al. (2004), and CS in both Lovejoy & Lemmon: Biver et al. (2016). Middle panel: $\delta ^{33}$S and $\delta ^{34}$S isotopic deviations measured in H_2_S, CS_2_, and OCS during two different periods at 67P together with the computed bulk value (Calmonte et al. 2017) and bulk sulfur isotope measurements together with individual impact craters in the returned Stardust sample from comet 81P/Wild 2 (Heck et al. 2012) in comparison to the mean ISM value (Chin et al. 1996). Top panel: $\delta ^{34}$S in OCS (Calmonte et al. 2017) amended by the 4 measured dust grains and corresponding bulk value from the Rosetta/COSIMA instrument at 67P/C-G (Paquette et al. 2017)

Figure 5 shows the collection of cometary sulfur isotope measurements obtained thus far in $\delta $-notation (per mil deviation from V-CDT) and compared to the mean ISM value (Chin et al. 1996). The measurements in the volatiles of 67P/C-G show a depletion predominantly in ^33^S. Calmonte et al. (2017) showed that neither mass-dependent nor mass-independent fractionation from photodissociation is responsible for the depletion in ^33^S and ^34^S. The authors also argued that the differences in the volatiles measured originate from different chemical pathways forming these molecules (cf. Sect. 4.2.2). In comparison, the depletion in $\delta ^{34}$S is within the 1-$\sigma $ error bars of the average ratio measured in the dust of 67P/C-G (Paquette et al. 2017).

Of the 24 analyzed impact craters in the dust impact residues on the aluminum foil returned by NASA’s Stardust mission, Heck et al. (2012) reported only one crater to differ in both $\delta ^{33}$S and $\delta ^{34}$S by more than 2-$\sigma $ from the V-CDT value. Looking at individual ratios there are 5 impact craters depleted by ≥2-$\sigma $ in $\delta ^{33}$S compared to one depleted in $\delta ^{34}$S. Generally, the scatter in $\delta ^{33}$S is larger compared to $\delta ^{34}$S and cannot be explained by the higher precision in $\delta ^{34}$S measurement alone. Nevertheless, the deviations from V-CDT in the 81P/Wild 2 data are small compared to the observations in 67P/C-G, pointing to either different processes and/or efficiencies in the isotope fractionation between volatiles and refractories. Another explanation is that the two comets formed at distinct locations in the early solar system and as a consequence from different source materials and at different temperatures (cf. also Sect. 4.1.1 on the D/H variation within the individual dynamical families of comets). In the case of 81P/Wild 2 Heck et al. (2012) concluded that most or all of the S-rich material formed in the solar system (see also Bullock et al. 2010; Tachibana and Huss 2005). Sulfur isotope ratios in 67P/C-G, on the other hand, are consistent with observations in the ISM (Chin et al. 1996) although the associated error bars are large. The degree of fractionation in 67P/C-G resembles presolar SiC grains from type II supernovae. Based on the Rauscher et al. (2002) 15 M_⊙_ supernova model, Hoppe et al. (2018) showed that a late supernova contribution could indeed reproduce the observed S-isotope anomaly. This again would point to a non-homogenized distribution of the material. Thus, in the early solar system the situation is complicated as the sulfur incorporated in 67P/C-G appears to originate from various sources.

#### Silicon Isotopes in Refractories and Their Implications for Origins

The ROSINA measurements showed a depletion in the heavy isotopes ^29^Si and ^30^Si with respect to the major isotope ^28^Si (Rubin et al. 2017). The origin of the atomic silicon was most likely sputtering by solar wind protons, which early in the mission still reached the silicate-rich surface of the nucleus (Bardyn et al. 2017; Wurz et al. 2015). ROSINA detected no silicon bound in molecules. The reported deviations at 67P/C-G from solar (McSween and Huss 2010) of 92.230% for ^28^Si, 4.683% for ^29^Si, and 3.087% for ^30^Si were $\delta ^{29}$Si = (−145 ± 98)$\permil $ and $\delta ^{30}$Si = (−214 ± 115)$\permil $ and contain 1-$\sigma $ errors (Rubin et al. 2017). Therefore, solar abundances could not be excluded (deviations between 1-$\sigma $ and 2-$\sigma $), however, several possible contributors to the depletion were put forward, including instrumental effects and fractionation in the sputtering process. On the other hand, together with the findings of other isotopes, a non-homogenized distribution of the material in the early solar system has to be considered too.

### Molecular and Elemental Ratios in Refractories and Their Implications for Origins

Bockelée-Morvan et al. (2000) compared relative abundances of volatiles at comet Hale-Bopp with those derived in interstellar ices, hot molecular cores, and bipolar outflows around protostars. From the resemblance of the volatiles’ abundance in these objects they concluded that similar chemical processes must be at work forming these compounds under comparable conditions, in particular grain-surface chemistry. In the vicinity of low-mass to massive protostars, i.e. from so-called hot corinos to hot cores, volatiles contained in these ice grain mantles evaporate and are then observed through their rotational lines using radio telescopes such as ALMA (Herbst and van Dishoeck 2009).

In the coma of 67P/C-G a multitude of molecules has been found by ROSINA (Le Roy et al. 2015) and in the near-surface environment of the comet by the lander mass spectrometers Ptolemy and COSAC (Goesmann et al. 2015; Wright et al. 2015). The combined analysis shows an inventory of volatiles, semi-volatiles, and refractories rich in organics (Altwegg et al. 2017b), in line with the findings in the cometary dust by the COSIMA mass spectrometer (Bardyn et al. 2017; Fray et al. 2016). Other key observations suggested the presence of ammonium salts in 67P/C-G (Poch et al. 2020; Quirico et al. 2016) and other comets (Mumma et al. 2017, 2018, 2019). Indeed, the mass spectra obtained by ROSINA DFMS (Altwegg et al. 2020; Hänni et al. 2019) showed all possible sublimation products of five different ammonium salts. Significant amounts of nitrogen may be bound in ammonium salts and hence hidden from observations due to high sublimation temperatures, which lead to the well-known deficiency of nitrogen in comets (Geiss 1987).

**Fig. 6:**
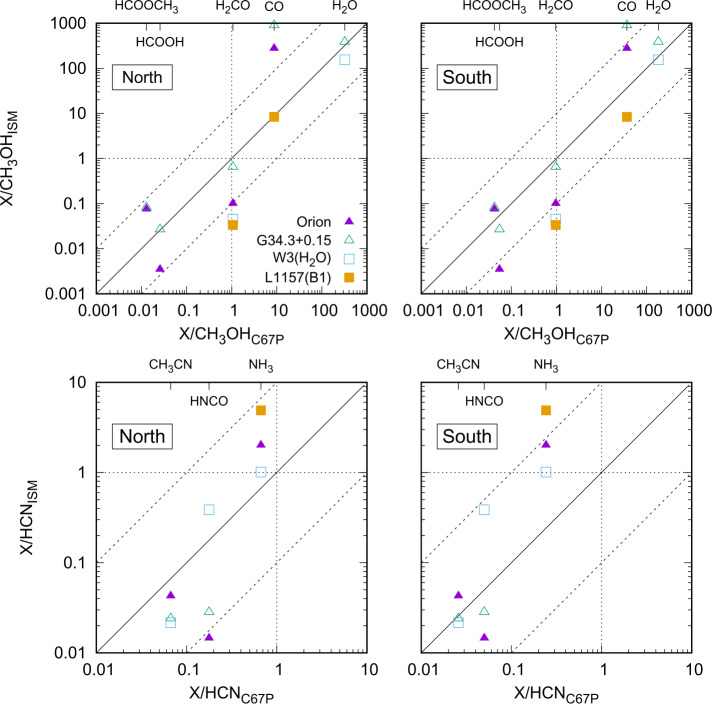
Relative abundances of oxygenated compounds with respect to CH_3_OH (top row) and N-bearing molecules with respect to HCN (bottom row) measured at comet 67P/C-G (x-axes, chemical formula on top) above the northern (left column) and southern (right column) hemispheres compared to relative abundances in the ISM (y-axes) after Bockelée-Morvan et al. (2000) and references therein (data from 67P/C-G: Le Roy et al. (2015), cf. Table 1). The solid black line denotes equal relative abundances while the two dashed lines represent deviations by a factor 10. Abundances equal to CH_3_OH and HCN are denoted by the vertical and horizontal dotted lines

Figure 6 shows a comparison of the relative abundances of oxygenated molecules (top row, normalized to methanol) and nitrogen-bearing molecules (bottom row, normalized to hydrogen cyanide) measured at comet 67P/C-G (x-axis) from Le Roy et al. (2015) and listed in Table 1 versus multiple objects in the ISM from Bockelée-Morvan et al. (2000) (y-axis). The measurements at 67P/C-G were performed above both the northern summer (left) and the southern winter hemispheres (right) at 3.1 au during the inbound part of the comet’s orbit, which led to different relative abundances in the coma of the comet. On the other hand, the derived ISM ice composition varies among the different objects, possibly affected by different temperatures. However, close trends in the relative abundances can be observed: Schuhmann et al. (2019) showed that relative abundances of hydrocarbons in 67P/C-G are similar to those derived from a model of gas-grain chemistry in a dark interstellar cloud (Hasegawa et al. 1992), including abundant amounts of unsaturated hydrocarbons (Altwegg et al. 2017b). A detailed comparison of comet 67P/C-G with low-mass protostar IRAS 16293-2422 B was very recently performed by Drozdovskaya et al. (2019) and revealed a close match between the two objects for a suite of CHO-bearing species. Correlations were also obtained for N- and S-bearing molecules, albeit with larger scatter. Similar conclusions were obtained for other comets (Mumma and Charnley 2011), which supports the suggestion that at least parts of the ices originated in the interstellar medium and that chemical alteration during and after incorporation into the comet was inefficient.

#### The O_2_ Story

One of the most surprising findings of the Rosetta mission was the detection of copious amounts of molecular oxygen, O_2_, by ROSINA (Bieler et al. 2015a). The measurements showed a strong correlation with water despite O_2_’s high reactivity and volatility compared to H_2_O. Other species of similar volatility, such as N_2_ and Ar, were found in much smaller amounts (cf. Sect. 3.3). Furthermore, the low abundances (or absence, cf. Taquet et al. (2018)) of O_2_ in star forming regions, e.g. in Orion (Goldsmith et al. 2011) and in the dense core of $\rho $ Ophiuchus A (Larsson et al. 2007; Liseau et al. 2012) are in contrast to the O_2_/H_2_O = 3.80 ± 0.85% measured at the comet. In the following, the presence of molecular oxygen in the coma of 67P/C-G has also been deduced from the OI 1356 Å/1304 Å ratio measured by the Rosetta Alice imaging spectrograph (Keeney et al. 2017). It was furthermore shown that a similar amount of O_2_ (Rubin et al. 2015b) is consistent with the measurements of the Neutral Mass Spectrometer (NMS; Krankowsky et al. (1986)) on board of the Giotto mission to comet 1P/Halley (Reinhard 1986). Even though NMS could not resolve O_2_ from species with close mass, i.e. methanol and atomic sulfur, the contribution of these species could be accounted for using an ion-neutral chemical network.

Several mechanisms for the formation of O_2_ have been discussed in the literature. Bieler et al. (2015a) proposed two scenarios favoring a primordial origin of the O_2_. The first is the formation of O_2_ and by-products through radiolysis or photolysis of water ice, similar to the Galilean moons Europa, Callisto, and Ganymede (e.g. Johnson et al. (2004) and Teolis et al. (2006)) where energetic ions from Jupiter’s magnetosphere irradiate the icy surfaces. However, typical radiolysis products H_2_O_2_ and HO_2_ in 67P/C-G were only found in low relative abundances comparable to $\rho $ Ophiuchus A (Bergman et al. 2011; Parise et al. 2012) or were even absent in the case of O_3_. The second possibility is the chemical formation of O_2_ and subsequent trapping in water ice during a phase of rapid cooling. Possible formation scenarios have since been refined and shall be discussed below. Furthermore, alternative formation scenarios have been investigated in the literature, namely, the dismutation of H_2_O_2_ during the desorption of water ice from the nucleus (Dulieu et al. 2017) and Eley-Rideal reactions of energetic water-group ions and neutrals (Yao and Giapis 2017). In both cases O_2_ is formed *in situ* and is not of primordial origin. The former scenario requires a high dismutation efficiency and the peroxide in the comet’s nucleus to be primordial. The latter scenario, as pointed out by Heritier et al. (2018), can only account for up to 10^−4^ of the observed O_2_ flux and furthermore the correlation of O_2_ with H_2_O is lost. For a more detailed discussion of *in situ* processes we refer to the review by Luspay-Kuti et al. (2018). Given that O_2_ has been detected unequivocally in 67P/C-G and tentatively in 1P/Halley, the question remains whether O_2_ is a common constituent in comets and how it formed.

Mousis et al. (2016b) showed that large amounts of O_2_ in the 1 – 10% range with respect to water can be formed through radiolysis of amorphous ice grains by galactic cosmic rays in low density environments such as the presolar cloud (cf. Zheng et al. 2006 and references therein). The authors also investigated the possibility of evaporation and re-trapping of volatile O_2_ in crystalline ices and clathrates upon entering the PSN. A late formation of O_2_ through irradiation in the midplane of the disk is difficult due to self-shielding of the disk material. Mousis et al. (2018a) therefore studied the impact of vertical transport of material from the midplane to the upper layers of the disk where the irradiation occurs. They conclude that even under favorable conditions the amount of O_2_ produced is some two orders of magnitude too low with respect to the abundances measured at 67P/C-G.

Another possibility, the irradiation from the decay of endogenic short- and long-lived radionuclides located inside the refractories in the nucleus, has been studied by Bouquet et al. (2018) based on the model by Mousis et al. (2017a) and a dust to ice ratio inside the nucleus of 4 (Rotundi et al. 2015). While sizeable amounts of O_2_ of up to 1% could be produced, other radiolysis products such as H_2_O_2_, are formed in amounts that are two orders of magnitude above the observed abundances.

Bieler et al. (2015a) also investigated the production of O_2_ after the formation of the nucleus. The penetration depth of cosmic rays forming O_2_ is on the order of a few meters which allows alteration of the uppermost layers of the comet. However, upon entry in the inner solar system after a close encounter with Jupiter in 1959 (Maquet 2015), the erosion of the comet’s surface is estimated to be several meters per 6.5-year orbit (Keller et al. 2015). The altered layer would have long since gone. On the other hand, if O_2_ is produced *in situ* through irradiation by solar wind, the O_2_/H_2_O ratio must exhibit variations associated to the plasma interaction of the comet with the solar wind. Bieler et al. (2015a), however, reported a stable O_2_/H_2_O ratio. As a result, a formation of O_2_ in the low density environment of the presolar cloud remains their most plausible scenario (Bieler et al. 2015a; Mousis et al. 2016b).

Taquet et al. (2016) used a model of the chemical evolution around an evolving star coupled to an astrochemical model for the gas phase and depth-dependent ice-grain chemistry (cf. Furuya et al. 2015). The considered O_2_ formation contains two main processes: first, neutral-neutral chemistry in the gas phase starting from abundant atomic O and molecular OH; and second, association reactions by atomic O recombination on or within the mantles of icy grains. Their model suggested that the formation of O_2_ through radiolysis and photolysis is inefficient as O_2_ is also converted to H_2_O through the recombination with H atoms.

Efficient formation of O_2_ (≥4% with respect to water ice) was achieved in high density regions with a lower gas-phase H/O ratios to decrease hydrogenation reactions (Taquet et al. 2017). Also, the measured amounts of O_2_ in the comet favor intermediate temperatures of the interstellar ices of ∼20 K. At such temperatures H atoms are efficiently sublimated and the increased mobility of O atoms on the grain surface enhances O association reactions. Furthermore, an increased cosmic ray ionization rate removes H from the solid phase.

In their simulations N_2_ and CO are formed later, on the outer layers of the icy grains. Thus the better correlation of O_2_/H_2_O (Bieler et al. 2015a) as opposed to N_2_/H_2_O (Rubin et al. 2018) and CO/H_2_O (Hässig et al. 2015) is a natural outcome of the model. On the other hand, the model predicted an N_2_/CO ratio of 50% in the ice, which is in contrast to the measurements at 67P/C-G (Rubin et al. 2015a). However, these outer layers of highly volatile species can be subject to evaporation upon heating during the transport from the dark cloud towards the protostellar disk. Subsequent recondensation is possible and the N_2_/CO ratio depends again on ice phase and temperature as discussed in Sect. 3.3.

Taquet et al. (2016) also investigated the production of O_2_ at the increased temperatures and UV fluxes expected during the protostellar collapse and protoplanetary disk formation. They find that O_2_ is formed on the per cent level with respect to water only in the upper layers of the disk. However, there the O_2_ seems rather associated to CO_2_ than H_2_O. Eistrup and Walsh (2018), on the other hand, found a sweet spot for the formation of O_2_ via ice grain chemistry in the presolar nebula from 120 – 150 au. Nevertheless, given that these results depend strongly on the chemical parameters of the involved interactions, the authors still favor a primordial origin of O_2_.

The third scenario investigated by Taquet et al. (2016) is the formation of O_2_ through luminosity outbursts of the low-mass host star, which could trigger warm gas-phase formation following the sublimation of water ice at temperatures above ∼100 K. However, these outbursts are short and hence the amount of O_2_ formed with respect to H_2_O remains <0.1%. The authors conclude that O_2_ must have a prestellar or molecular cloud origin.

In laboratory experiments, O_2_ in water ice (Ioppolo et al. 2008; Miyauchi et al. 2008) is efficiently hydrogenated at low temperatures to form H_2_O and H_2_O_2_. This is in conflict with the measured amounts of O_2_ and the low H_2_O_2_/O_2_ (as well as HO_2_/O_2_) ratio of 10^−3^ in 67P/C-G from Bieler et al. (2015a). Mousis et al. (2016b), on the other hand, showed that O_2_ can be efficiently incorporated and stabilized into the structure of crystalline ices.

Also, Laufer et al. (2018) showed that desorption fluxes of O_2_ from amorphous water ice are much higher compared to other highly volatile species such as N_2_ and Ar, even if the initial relative abundances in the gas phase before trapping were comparable. This could in part explain the difference in the abundance of these volatiles. Furthermore, their results also indicated that only 10 – 20% of the O_2_ is released in the kinetic temperature range of 65 – 120 K derived for Orion (Goldsmith et al. 2011). At comet 67P/C-G the O_2_ is correlated and hence co-desorbing with water (Bieler et al. 2015a) at a higher temperature compared to Orion. This further complicates matters and more work on the stability of O_2_ in temperature ranges representative for the comet are required to obtain solid constraints for this problem.

#### Sulfur Chemistry

Sulfur in dense cloud cores has been found to be depleted by roughly two orders of magnitude compared to the diffuse interstellar medium (Ruffle et al. 1999). Such a depletion is unique to sulfur (Drozdovskaya et al. 2018) and the missing sulfur has not been conclusively identified (e.g. Bilalbegović and Baranović 2014). Measurements at comet 67P/C-G revealed a wealth of sulfur-bearing volatiles (Calmonte et al. 2016). Sulfur has also been detected in the refractory phase (Paquette et al. 2017). Thus, the link from the diffuse interstellar medium, through the dense clouds, all the way to comets contains crucial links to the formation and processing of the material through the different evolutionary stages.

Formation of S-bearing volatiles have been attributed to ice grain chemistry: for instance, Ruffle et al. (1999) explained the formation of H_2_S by the adsorption of positively charged S^+^ and HS^+^ ions on negatively charged ice grains and subsequent hydrogen attachment. A suite of laboratory experiments have shown that H_2_S can then be transferred into other S-bearing molecules such as OCS and CS_2_ in the presence of C-bearing species such as CO in the ice (see Calmonte et al. (2016) and references therein). In addition, the presence of other organosulfurs point to the importance of ice/dust-grain chemistry. Ice grain chemistry and radiolysis of H_2_S (Mousis et al. 2017b) and other sulfur-bearing species can form S-polymers up to the stable ring molecule S_8_, out of which the possible fragments S_2_, S_3_, and S_4_ have been identified at 67P/C-G. For S_2_ two distinct populations have been suggested: first, S_2_ in volatile form and second, S_2_ as a dissociation product from larger S-bearing molecules up to solid S_8_ associated to hot dust grains in the coma near-perihelion.

This S-residue formed by radiolysis and photolysis of interstellar ices is difficult to observe with radio astronomy. Its formation has been hypothesized to be the main cause for the observed depletion in dense cores, even though the model by Woods et al. (2015) suggested a contribution of up to 6% of the missing sulfur only. Nevertheless, the sulfur molecules S_2_, S_3_, and S_4_ detected in the coma of comet 67P/C-G would support this theory. However, absolute abundances of these molecules in the refractory part could not be derived due to the strong temperature dependence of the outgassing. Furthermore, the mass range of the ROSINA DFMS sensor did not include the parent S_8_.

**Fig. 7:**
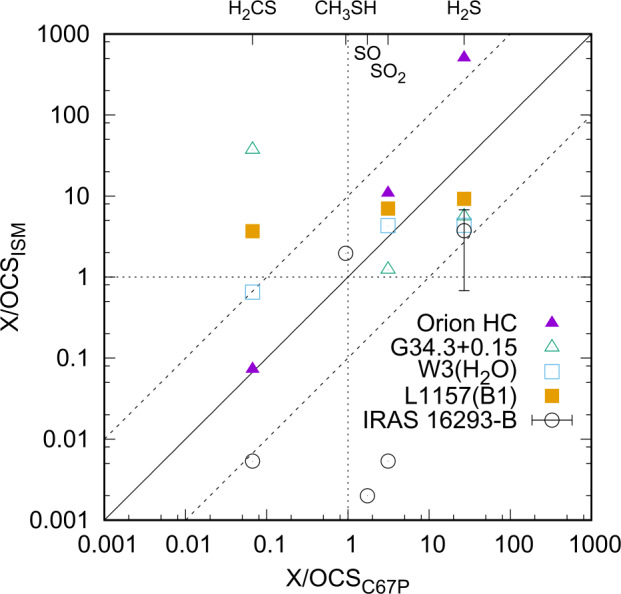
Similar to Fig. 6. Relative abundances of sulfur-bearing compounds normalized to OCS measured at comet 67P/C-G (x-axis) from the bulk abundances in Table 2 from Calmonte et al. (2016) compared to relative abundances in the ISM (y-axis) from Drozdovskaya et al. (2018) and Bockelée-Morvan et al. (2000) with references therein

Bulk abundances of the volatile S-bearing species, on the other hand, have been obtained (Calmonte et al. 2016). Figure 7 shows relative abundances of S-bearing volatiles in the comet versus various protostars (see Bockelée-Morvan et al. (2000) and Drozdovskaya et al. (2018)). Again, the spread among protostars can be considerable but with a few exceptions the trends seem similar to the observed cometary abundances. Furthermore, the recent study by Drozdovskaya et al. (2019) shows a good correlation of the sulfur-bearing species in comet 67P/C-G with low mass protostar IRAS 16293-2422B.

The presence of S_2_ in the ices of the comet led Calmonte et al. (2016) to the conclusion that the ices of the comet predate the formation of the solar system. The reason for this is the very short lifetime of the sulfur dimer in the vapor phase when exposed to UV radiation. Mousis et al. (2017b) then also discussed a post solar system formation scenario where S_2_ forms from H_2_S in ice grains that condensed in the protosolar nebula as long as they are sufficiently irradiated in the upper layers of the disk. Independent of the pre- or protosolar nebula origin, the radiolysis formation model predicts correlated outgassing of S_2_ and H_2_S. Furthermore, the radiation also forms voids in amorphous H_2_O ice in which S_2_ can accumulate and remain stable even after crystallization. This suggests a correlation also between S_2_ and H_2_O (Mousis et al. 2018b), while at 67P/C-G the correlation among these species was rather poor (Calmonte et al. 2016).

## Post-Formation Evolution of 67P and Other Comets

A’Hearn et al. (2012) investigated the relative abundances of the major volatiles CO/CO_2_/H_2_O in a multitude of comets. Any differences among the dynamical groups of comets, i.e. among Kuiper belt and Oort cloud comets, might link them to not only their formation location (Mumma and Charnley 2011) but also to their post-formation evolution. Several post-formation scenarios are discussed in the following.

### Loss of Highly Volatile Molecules in 67P Through Evolutionary Processes?

The noble gases argon, krypton, and xenon have been identified in the coma of comet 67P/C-G (Balsiger et al. 2015; Marty et al. 2017; Rubin et al. 2018). 67P/C-G proved to be enriched in xenon over krypton and krypton over argon in comparison to the early Sun (Lodders et al. 2009), i.e. ^84^Kr/^36^Ar_67P/C-G versus Sun_ = 144 ± 58 and ^132^Xe/^36^Ar_67P/C-G versus Sun_ = 683 ± 279 and solar wind fluxes from the Genesis mission (Meshik et al. 2014), i.e. ^84^Kr/^36^Ar_67P/C-G versus SW_ = 139 ± 32 and ^132^Xe/^36^Ar_67P/C-G versus SW_ = 291 ± 72. For the noble gas neon only an upper limit could be derived. When comparing expected ratios of noble gases trapped in amorphous water ice (Bar-Nun et al. 1988; Dauphas 2003) it is not possible to match both ratios at the same time, ^132^Xe/^36^Ar_67P/C-G_ and ^84^Kr/^36^Ar_67P/C-G_. Similar issues, as discussed in Sect. 3.3, are raised for other types of ices including different crystalline ices formed in the protosolar nebula.

So, while comets belong to the most pristine objects in the solar system (Geiss 1987), post-formation alteration of the budget of highly volatile species cannot be excluded.

#### The Centaur Stage

One possibility is evolutionary processing occurring after the comet’s formation, possibly through the preferential loss of highly volatile species during the comet’s journey from the Scattered disk, through several Myr as a Centaur (Guilbert-Lepoutre et al. 2015), to the inner solar system (Maquet 2015). This is based on the idea that a dynamical cascade between Kuiper belt objects, Centaurs, and JFCs exists (Levison and Duncan 1997; Tiscareno and Malhotra 2003) which is different from the dynamical history of Oort cloud comets (Weissman et al. 2020).

**Fig. 8:**
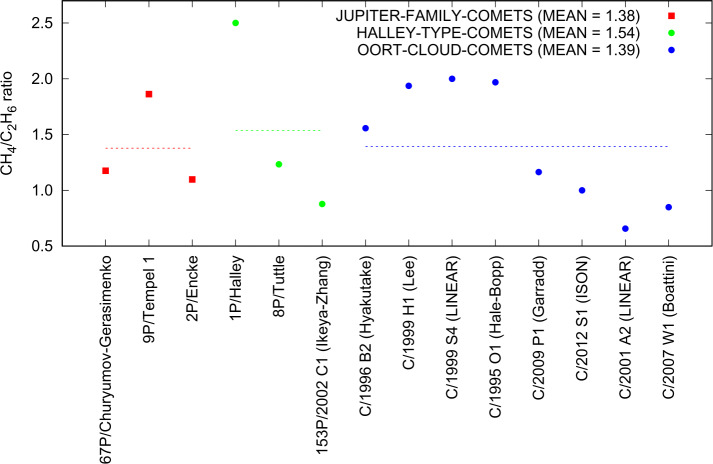
CH_4_/C_2_H_6_ ratio in several comets including 67P/C-G. Strong variations within each family (JFCs, Halley-type comets (HTCs), and Oort cloud comets) are observed. References and heliocentric distance of the observations: 67P/C-G (1.5 au): Schuhmann et al. (2019), Tempel 1 (1.5 au), Encke (1.2 au), Lee (1.1 au), Ikeya-Zhang (0.8 au), and Hale-Bopp (0.9 au): Dello Russo et al. (2016) and references therein, S4 (LINEAR) (0.8 au), ISON (1.1 au), A2 (LINEAR) (1.2 au), and Boattini (0.9 au): Lippi et al. (2020), Halley (0.9 au): Eberhardt (1999); Garradd (1.6 au): Paganini et al. (2012), and Tuttle (1.2 au): Böhnhardt et al. (2008)

According to Guilbert-Lepoutre et al. (2016), a comet such as 67P/C-G has its surface thermally altered down to a few hundred meters, where the temperature reaches up to 80 K. Depending on the orientation of the rotation axis even a transition of amorphous to crystalline ice may occur. In comparison, the erosion of 67P/C-G’s surface on its current orbit is estimated to be a few meters per revolution (Keller et al. 2015). The comet reached the inner solar system after a close encounter with Jupiter in 1959 (Maquet 2015). If indeed the comet has not been that close to the Sun before 1959, the number of perihelion passages in the inner planetary system, which occur every ∼6.5 years, is limited and hence today’s outgassing might still occur from a layer thermally altered during the Centaur stage. In comparison to Oort cloud comets, Schuhmann et al. (2019) find rather low abundances of the two simplest alkanes (CH_4_ and C_2_H_6_) relative to water in 67P/C-G, and point out that other highly volatile species are also low including N_2_ and CO. Furthermore, as shown in Fig. 8, the ratio of CH_4_ to the lesser volatile C_2_H_6_ varies greatly among comets but is also low in 67P/C-G.

Nevertheless, more work is required in this respect. Nucleus models by Prialnik (1992) predict lower penetration depths of the heat wave of a few tens of meters due to the low thermal conductivity of cometary material and hence the presence of amorphous ices within tens of meters from the surface. On top of that there is only limited information on the temperature-dependent mobility of highly volatile species (Lauck et al. 2015) in a highly porous icy and refractory material relevant to comet 67P/C-G.

#### Thermal Evolution Through Multiple Perihelion Passages

Evolutionary processes and compositional differences among the different cometary families have been studied before, including the thermal evolution of the interior of comets and associated outgassing (e.g. Keller et al. (2015)). The numerical model by Prialnik et al. (2004) predicts the build-up of a dust layer affecting the outgassing behavior of the comet. Furthermore, also the volatiles recede to different depths based on their volatility (De Sanctis et al. 2006; Gortsas et al. 2011; Marboeuf and Schmitt 2014). However, Fulle et al. (2016) pointed out that numerical models exhibit problems reproducing observed outgassing profiles as a function of heliocentric distance.

A’Hearn et al. (2012) investigated the abundances of the volatile species H_2_O, CO_2_, and CO in comets. The results showed a wide range of abundance ratios in both JFCs and LPCs/HTCs. The authors concluded that this is unlikely the result of evolution through successive perihelion passages. Also, trends within comet families were not entirely defined, but it is clear that the volatile abundances have largely overlapping ranges in all the dynamical families. This suggests a largely overlapping region of origin, similar to the conclusion from the D/H measurements in cometary water (Sect. 4.1.1). With a two-order-of-magnitude spread in CO/H_2_O, there is some indication that LPCs show higher values of CO/H_2_O and that JFCs exhibit somewhat lower values. It cannot therefore be concluded whether these compositional differences are the result of a different location of formation and/or subsequent thermal evolution, including as a Centaur in the case of JFCs (cf. Sect. 5.1.1).

#### Collisional Heating of Cometesimals

Several of the comets visited to date show distinct bi-lobate shapes (Sunshine et al. 2016). These objects and their precursors can form through hierarchical accretion or collisionally induced disintegration of larger parent bodies. Jutzi and Asphaug (2015) investigated comets like 67P/C-G forming in sub-catastrophic collisional mergers leading to bi-lobate shapes and also the layering that has been observed in 67P/C-G (Massironi et al. 2015). There is still a debate as to how primordial these objects are (Davidsson et al. 2016; Fulle et al. 2016; Weissman 1986). Jutzi and Asphaug (2015) showed that the low density and high porosity can be preserved in such collisions, however, they point out that Kuiper belt objects larger than 5 km undergo multiple shape changing (Jutzi et al. 2017) and also catastrophic disruptions (Jutzi and Benz 2017) throughout the lifetime of the solar system. While the details of the origin of 67P/C-G and other comets are discussed in the accompanying paper by Weissman et al. (2020) we shall here focus on the presence of highly volatile species in these objects. This includes the gases of CH_4_, CO, and others found in the comae of many comets (Bockelée-Morvan et al. 2004; Mumma and Charnley 2011). Hence, Schwartz et al. (2018) investigated the amount of impact heating caused by catastrophic collisions. Their results suggest that the reaccumulated comet is only marginally heated, i.e. <1 K, while material heated-up by several tens of K in sizeable amounts, i.e. up to 10%, can only be found in the ejecta. As a consequence, highly volatile species can be retained in cometary ices, even after a violent encounter. Hence the presence of highly volatile molecules and 67P/C-G’s low density (Pätzold et al. 2016) alone is not proof for the nucleus itself to be primordial. Nevertheless, Jutzi et al. (2017) predict that a comet like 67P/C-G undergoes many shape changing collisions, critically depending on the differential size distribution of the objects in the disk. Davidsson et al. (2016) prefer a shallow size index, which reduces the number of collisional interactions of cometesimals and hence increases the survival rate in their primordial form. Jutzi and Benz (2017), on the other hand, prefer a steeper size distribution and the final object is hence the result of many collisions or could even form from the ejected material itself.

#### Impact of Radiogenic Heating on Comets

Mousis et al. (2017a) investigated the consequences of the radioactive decay of ^26^Al and ^60^Fe on the budget of highly volatile molecules such as N_2_ and CO in the comet. A thermal nucleus model has been employed to simulate heat transfer, phase transition and sublimation of ices, and the diffusion of volatiles through the porous material of the comet. The results depend on several critical parameters including the initial size of the object, the dust to ice ratio in the nucleus, and the initial abundance of radioactive isotopes, in particular the controversial amounts of ^26^Al as Prialnik and Podolak (1995) pointed out. Nevertheless, their results suggested that either the formation of comet 67P/C-G was delayed by several million years with respect to the formation of Calcium-Aluminum-rich Inclusions in the PSN or the accretion time of the nucleus was on the order of a few million years to prevent formation of large devolatilized volumes inside the nucleus. Prialnik and Podolak (1995), on the other hand, reported that objects smaller than 20 km are mostly unaltered in the presence of the longer lived ^40^K, ^232^Th, ^235^U, and ^238^U radioisotopes as long as the initial amounts of ^26^Al are negligible. The addition of ^26^Al reduces the critical radius of an object for crystallization to occur and also the time of its onset after formation is shorter. During crystallization highly volatile molecules, such as CO, are released before condensing again in the colder regions farther from the center of the comet. Heat advection by the flow of volatile species may even surpass the heat conduction of the solid phase in such a case. It is thus clear that an increase in the temperature resulting from any kind of radiogenic heating critically affects the composition of a comet.

## Summary and Conclusions

The origin of the material in comets has been a main focus of research for a long time. The recent Rosetta mission provided numerous new insights to test contemporary theories and to verify our current understanding of the major processes that are involved.

- After the first measurement of the HDO/H_2_O ratio in a JFC (i.e. Hartley 2) it was hypothesized that there is an intrinsic difference between Oort cloud and Kuiper belt/Scattered disk objects (Hartogh et al. 2011). This rekindled the discussion on the Earth’s water being of cometary origin. However, recent measurements of elevated D/H ratios in the water of additional JFCs, including 67P/C-G, countered this theory (Altwegg et al. 2015; Biver et al. 2016; Paganini et al. 2017). Thus, it seems unlikely that comets from the Kuiper belt and beyond were the major source for the water on Earth. Instead, the results suggest that the D/H ratio reflects the formation location of comets rather than where they are found today: comets formed over a wide range of heliocentric distances and were relocated afterwards. Both lobes of 67P/C-G share the same D/H ratio within error bars. This hints at a common location of formation before merging (Schroeder et al. 2019).
- Whether the ice itself is inherited from the ISM is more difficult to assess from the HDO/H_2_O ratio alone. Comparing the D/H ratio from both HDO/H_2_O and D_2_O/HDO seems to be the better measure (Furuya et al. 2016). The very different ratios in 67P/C-G indicate that at least parts of the ice must be inherited from the cold temperature chemistry occurring in the presolar cloud (Altwegg et al. 2017a) and that subsequent isotope-exchange reactions were not efficient.
- Apart from D/H, other isotopes in the volatile material of many comets show marked deviations from solar system bulk ratios. In particular the nitrogen isotopes are not yet understood. The three molecules observed thus far, CN, HCN, NH_2_, show a consistent enrichment of ^15^N in all comets for which we have measurements (Hily-Blant et al. 2017) whereas much larger variations are found in the ISM (Füri and Marty 2015 and references therein). Furthermore the isotopes of the volatile sulfur (Calmonte et al. 2017), oxygen (Hässig et al. 2017; Schroeder et al. 2018), xenon (Marty et al. 2017), and refractory silicon (Rubin et al. 2017) show or at least hint at deviations from bulk solar system abundances. Other volatiles, e.g. argon and krypton (Balsiger et al. 2015; Rubin et al. 2018) and refractory sulfur (Paquette et al. 2017) are within the error bars of solar system bulk abundances. The refractories in other comets, including the returned samples from comet Wild 2, contain thermally processed material from the inner solar system with only small amounts of presolar material. Thus, radial mixing occurs, but the protoplanetary disk remains only partially homogenized. Therefore, the isotopic and possibly also elemental and molecular abundances in at least the icy phase at the location of the comet’s formation differed from bulk solar system.
- Comets may contain significant amounts of ammonium salts (Mumma et al. 2019) that are likely to account for the to-date observed deficiency of nitrogen in comets (Altwegg et al. 2020; Geiss 1987; Poch et al. 2020; Quirico et al. 2016).
- The relative abundances of the volatiles in comets exhibit similarities to the ISM (Bockelée-Morvan et al. 2000) and comet 67P/C-G is no exception (Drozdovskaya et al. 2019). Also the large amounts of unsaturated hydrocarbons and other organics (Altwegg et al. 2017b; Schuhmann et al. 2019) are consistent with the ISM expectations (Hasegawa et al. 1992). This further solidifies an ISM heritage of the volatile material in 67P/C-G. The complexity of the organic material found in comets is larger than previously known and requires time to form.
- Similar conclusions arise from the abundant amounts of O_2_, which were well correlated to H_2_O in 67P/C-G (Bieler et al. 2015a). O_2_ has a presolar or molecular cloud origin and formed either through cold temperature chemistry (Taquet et al. 2017) and/or radiolysis (Mousis et al. 2016b). Chemical formation requires slightly increased formation temperatures (∼20 K) and gas densities ($\text{n}_{\mathrm{H}} \gtrapprox 10^{5}~\text{cm}^{-3}$), similar to the case of the dense core $\rho$ Ophiuchus A, but warmer compared to the lower mass environment expected for the early Sun. On the other hand, radiolysis and photolysis of water ice as a formation scenario for O_2_ leads to the formation of byproducts such as H_2_O_2_, HO_2_, and O_3_ in amounts much larger compared to that observed at 67P/C-G.
- The presence of S_2_, which has a very short lifetime when exposed to UV photons, requires preservation in the ices originating in the presolar cloud (Calmonte et al. 2016; Mousis et al. 2017b). However, the degree to which this also applies to other volatiles, apart from H_2_O, O_2_, and S_2_ (Bar-Nun et al. 2012; Greenberg et al. 2017), or whether they were evaporated and then recondensed from the gas phase in the protosolar nebula (Mousis et al. 2018b) is still debated. It seems clear, however, that a single ice phase is not able to reproduce the observed relative abundances, and/or the material in the comet was subject to evolutionary processes, such as temperature-dependent fractionation, occurring on different time scales of the comet’s journey through the solar system.
- Numerous molecular species are associated with life as we know it (Seager et al. 2016) but have an abiotic origin in 67P/C-G and other comets. Aside from O_2_ this includes organohalogens (Fayolle et al. 2017), glycine, phosphorous (Altwegg et al. 2016) and various organo-sulfurs (Calmonte et al. 2016). Even if not for the water, comets could indeed have delivered sizeable amounts of material to the Earth. A contribution of $22 \pm 5$% to the terrestrial atmosphere was derived based on the xenon isotopic composition of 67P/C-G compared to the terrestrial atmosphere (Marty et al. 2017). As a consequence, also copious amounts of organic species would have found their way to the inner solar system and the Earth (Marty et al. 2016; Rubin et al. 2019b).
